# Exploring the thermal behaviour of the solvated structures of nifedipine

**DOI:** 10.1107/S2052520623001282

**Published:** 2023-03-09

**Authors:** Eleanor C. L. Jones, Kate E. Goldsmith, Martin R. Ward, Luis M. Bimbo, Iain D. H. Oswald

**Affiliations:** a Strathclyde Institute of Pharmacy and Biomedical Sciences, University of Strathclyde, 161 Cathedral Street, Glasgow, G4 0RE, United Kingdom; b Department of Pharmaceutical Technology, Faculty of Pharmacy, University of Coimbra, Coimbra, Portugal; c Centre for Neuroscience and Cell Biology and Centre for Innovative Biomedicine and Biotechnology, University of Coimbra, Coimbra, Portugal; CSIR–National Chemical Laboratory, India

**Keywords:** solvate, desolvation, nifedipine, pharmaceutical, variable-temperature X-ray diffraction

## Abstract

Understanding the solvation and desolvation of pharmaceutical materials is an important part of materials discovery and development. *In situ* structural data are vital to understand the changes to crystal form that may occur in the system. In this study, the isolation and characterization of seven solvates of the L-type calcium channel antagonist, nifedipine, is described using variable-temperature powder X-ray diffraction so that the structural evolution as a function of temperature can be followed. The solvates reported herein can be split into those that are structurally similar to the previously reported dimethyl sulfoxide (DMSO) and dioxane solvates and those that have a novel packing arrangement. Of particular note is the solvate with tetrahydrofuran (THF) which has a hydrogen-bonding motif between the nifedipine molecules very similar to that of metastable β-nifedipine. In addition to variable-temperature X-ray diffraction, the stability of the solid forms was assessed using differential scanning calorimetry and thermogravimetric analysis and indicates that in all cases desolvation results in the thermodynamically stable α-polymorph of nifedipine even with the THF solvate. From the diffraction data the pathway of desolvation during heating of the DMF solvate showed conversion to another likely 1:1 polymorph before desolvation to α-nifedipine. The desolvation of this material indicated a two-stage process; first the initial loss of 90% of the solvent before the last 10% is lost on melting. The methanol solvate shows interesting negative thermal expansion on heating, which is rarely reported in organic materials, but this behaviour can be linked back to the winerack-type hydrogen-bonding pattern of the nifedipine molecules.

## Abstract 2

The formation of nifedipine solvates is explored and the desolvation pathways probed through a combination of variable-temperature X-ray powder diffraction, differential scanning calorimetry and thermal gravimetric analysis.

## 1. Introduction

The screening process of pharmaceutical materials is an important step in understanding their solid form landscape. As part of this, recrystallization of an active pharmaceutical ingredient (API) from different solvents is commonly performed which often results in the isolation of a solvated solid form of the API. This is typically not desirable as solvates are prone to desolvate/convert form during storage and secondary processing that can result in deterioration of the intended pharmaceutical formulation. For example, levothyroxine is commercially used in its pentahydrate form (LSP), to treat hypothyroidism. However, LSP is susceptible to dehydration to the monohydrate form when stored under elevated temperature and low relative humidity; such instability is undesirable (Shah *et al.*, 2019 ▸). This product has been known to be recalled due to issues with chemical and physical stability; however, since there is no alternative treatment available, the instability needs to be addressed (Kaur & Suryanarayanan, 2021 ▸). Therefore, there is a requirement to study and understand the formation and collapse of solvated materials alongside their properties so that the risks of their use can be mitigated against.

There have been a number of pharmaceutical solvates that have been thoroughly investigated, including sorafenib tosyl­ate, ciclesonide, olanzapine, gallic acid and trimesic acid, and even solvated co-crystals (Yang *et al.*, 2019 ▸; Zhou *et al.*, 2016 ▸; Bhardwaj *et al.*, 2013 ▸; Braun *et al.*, 2013 ▸; Ward & Oswald, 2020 ▸; Bodart *et al.*, 2021 ▸; Liu *et al.*, 2019 ▸; Marjo *et al.*, 2011 ▸; Moreno-Calvo *et al.*, 2011 ▸; Nowak *et al.*, 2022 ▸). The stability of solvates is a major consideration such that in some cases it is problematic but in others can be used to improve the physical properties of the material, similar to cocrystallization. For example, the ethanol solvate of Pranlukast (a drug used in the treatment of asthma) has been shown to be four times more soluble than the stable hemi-hydrate form. However, the ethanol solvate transforms to the hemi-hydrate at 90% relative humidity which limits the ability for the more soluble solvate to be taken forward in development (Furuta *et al.*, 2015 ▸). There have been a number of desolvation studies that have tried to resolve and understand solvate formation and the structural relationships between the solvate and the polymorphs of the desolvated product (Aitipamula *et al.*, 2011 ▸; Minkov *et al.*, 2014 ▸; Yang *et al.*, 2019 ▸). Olanzapine solvates have been extensively studied by Bhardwaj *et al.* (2013 ▸). In their study, a relationship between the packing of 18 isomorphous solvates and the pure thermodynamically stable form I was established which helped to rationalize its formation during desolvation (Bhardwaj *et al.*, 2013 ▸). In addition, crystal lattice energy calculations indicated that olanzapine is not able to form dense packing arrangements, but the common layered structure enables it to be a prolific solvate former. A further study of aripiprazole showed that the hydrogen-bonding pattern of the API in the solvate crystal structure can be retained on desolvation to produce a specific polymorph. Using the structures from three solvates (methanol, ethanol and di­chloro­methane), form III was reproducibly isolated due to the dimer hydrogen-bonding motif common to all structures (Braun *et al.*, 2009 ▸). But despite these instances of projecting the outcome of a desolvation based on the packing of the solvate and the pure forms, it is not always possible to do so (Aitipamula *et al.*, 2011 ▸). It is clear that in order to gain a better understanding of desolvation processes and be able to use this routinely to access new forms, *in situ* structural measurements are required to follow the pathway of structural changes that occur.

In this paper, we aim to assess the potential relationship between solvated crystal structure and resulting desolvated forms using a known pharmaceutical compound, nifedipine [3,5-di­methyl 2,6-di­methyl-4-(2-nitro­phenyl)-1,4-di­hydro­pyridine-3,5-di­carboxyl­ate, NIF, Scheme 1]. We investigated the solvation of NIF in a range of 18 solvents and subsequent desolvation as a method to access metastable forms. The metastable polymorphs (β to δ; Table 1 ▸) of NIF have only been isolated from the melt which indicates that these forms are sufficiently stable for identification under ambient conditions. In this study, we have been able to identify novel solvates and track their desolvation through variable-temperature X-ray diffraction (single-crystal and powder; VT-XRPD) enabling the structural characterization as a function of heating. The structural work is supplemented with thermogravimetric analysis–differential scanning calorimetry (TGA/DSC) to support the crystallographic observations. 
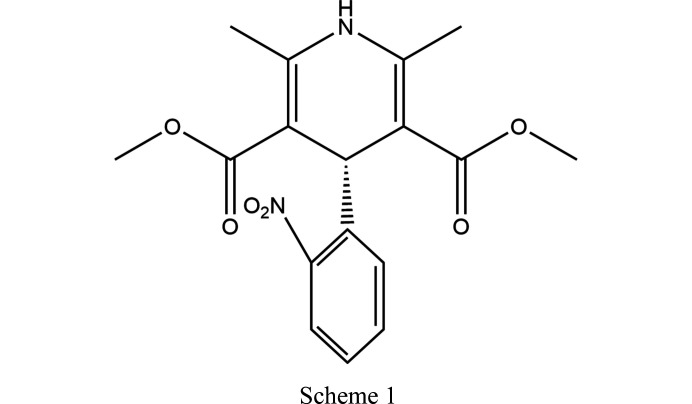


## 2. Experimental

### 2.1. Materials

An excess of NIF (Alfa Aesar, purity 98%) was suspended and slurried in selected solvents (1 g ml^−1^) for 24 h using a magnetic stirrer to agitate the slurry (∼295 K). A list of solvents used in this study can be found in Table S2 in the supporting information. Solid material was separated from the solution using a centrifuge and dried between filter paper before analysis. The supernatant solution was filtered using a 0.2 µm PTFE filter into clean glass vials and covered with pierced lids to aid slow evaporation of the solvent and obtain single crystals for analysis. NIF decomposes when exposed to light, therefore vials were wrapped in aluminium foil to minimize exposure to light and stored in a fumehood until suitable crystals were obtained.

### 2.2. Single-crystal diffraction

X-ray diffraction data of α-NIF, recrystallized from ethanol, were collected using a Bruker D8 Venture diffractometer with IμS Microfocus Source (Cu *K*α1, λ = 1.54178 Å) and Photon II detector at 297 K. Data were reduced using *Apex 3* software, incorporating *SAINT* (V8.40B) (Bruker, 2012 ▸). *SADABS* was used for absorption correction (Sheldrick, 1996 ▸). Samples were cooled using an Oxford Cryosystems Cryostream 800 system (Cosier & Glazer, 1986 ▸). The following solvated structures were collected on a Bruker Kappa APEX II with IμS Microfocus Source (Mo *K*α1, λ = 0.71073 Å) and APEX II CCD detector at 100 K, with the exception of di­methyl­acetamide (DMA) which was collected at 173 K (due to a phase transition below this temperature): pyridine, morpholine, tetra­hydro­furan, di­methyl­form­amide, di­methyl sulfoxide, methanol. Data were reduced using similar procedures as above. For the low temperature behaviour of the methanol solvate, variable-temperature data of a single crystal of the methanol solvate were collected in 25 K increments from 100 to 275 K using an Oxford Cryosystems Cryostream 800 system (Cosier & Glazer, 1986 ▸). Diffraction data for the 1,4-dioxane solvate were collected using an Rigaku Oxford Diffraction system (Synergy-I diffractometer (Cu *K*α, λ = 1.54184 Å) with a hybrid pixel array detector) at 100 K. Data were reduced using *CrysAlis PRO* with SCALE 3 ABSPACK correction implemented (Rigaku Oxford Diffraction, 2021 ▸).

All structures were solved using *ShelXT* (Sheldrick, 2015*b* ▸) intrinsic phasing incorporated in the *Olex2* software (Dolomanov *et al.*, 2009 ▸). Refinements were carried out using *ShelXL* (Sheldrick, 2015*a* ▸) least-squares refinement. Non-hydrogen atoms were anisotropically refined before the addition of hydrogen atoms in geometrically-calculated positions. Three structures showed disorder in the solvent molecules: tetra­hydro­furan, morpholine and di­methyl­acetamide. To model these, the occupancy of morpholine and tetra­hydro­furan atoms were set at 0.5 through the use of the ‘Split’ function in *Olex2*. The atoms in the second component were moved to positions where residual electron density was highest. The atoms were being modelled around an inversion centre and so we decided to model the whole molecule at 0.5 occupancy rather than half the molecules at occupancy of 1; this also helped us to create the z-matrix for refinement against our powder diffraction data. The atoms in the di­methyl­acetamide molecule were split using the same procedure, with atoms refined before fixing the occupancy to 0.85 in one molecule and the remaining molecules had occupancy set to 0.15. Distances between atoms were restrained with the SADI atoms, *DFIX* restraint, and EADP constraints applied. All crystallographic data are located in the supporting information (Table S3).

### 2.3. X-ray powder diffraction

Screening of the X-ray powder diffraction patterns of the samples was carried out using a Bruker D8 Advance II powder diffractometer with a multi-well flat plate, Cu *K*α1 X-ray source (λ = 1.5406 Å) at 297 K. An angular range of 4–35° 2θ with 0.017° 2θ step size with one second exposure per step was used. Excess solvent was removed from samples using filter paper prior to placing on a 28-well plate constructed from steel with Kapton film backing. From these data, samples of interest were identified and subject to further data collection in capillary transmission geometry as described below.

### 2.4. Variable-temperature X-ray powder diffraction

Solids from the slurry experiments were lightly ground to break up any aggregates using an agate pestle and mortar and transferred to 0.7 mm diameter borosilicate glass capillaries. A Bruker D8 Advance diffractometer with Johansson monochromator (Cu *K*α1, λ = 1.5406 Å) was used to collect the data. Samples were heated in 5 K increments (360 K min^−1^) using an Oxford Cryosystems Cryostream 800 system (Cosier & Glazer, 1986 ▸) and held at the target temperature for 5 min before commencing the data collection (4–35°, 2θ, 0.017° per step, one second exposure). For the samples 1,4-dioxane and tetra­hydro­furan, the heating process took 30 min to increase from 293 K to the next temperature in the series; 343 and 313 K, respectively. Material isolated from pyridine and methanol slurries provided XRPD data consistent with that of α-NIF, therefore single crystals of these solvates were grown and ground for capillary XRPD data collection. In all cases, unit-cell parameters were determined over the range of temperatures for each solvated structure using Rietveld refinement through the *Topas* software in the batch processing sequential mode (*Topas Academic* V5) (Coelho, 2018 ▸). The fitting of the patterns near the transition were revisited due to the automated procedures not coping with the multiple phases and the unit-cell parameters used in the plots.

For the low-temperature phase of the DMA solvate, the powder pattern (100 K data) was indexed using DICVOL06 (Boultif & Louër, 2004 ▸) in *DASH* (David *et al.*, 2006 ▸) to give unit-cell parameters, *a* = 7.83743 (13) Å, *b* = 12.6144 (2) Å, *c* = 13.2163 (3) Å, α = 62.9281 (19)°, β = 74.129 (2)°, γ = 64.8735 (17)°. From these parameters, the content of the asymmetric unit was calculated to be one molecule of NIF and one molecule of DMA. The structure was solved from the X-ray powder diffraction data using simulated annealing (*DASH*; David *et al.*, 2006 ▸). Rietveld refinement of the new polymorph against the low-temperature powder data was performed using *Topas Academic* (*R*_wp_ = 3.85%). The refinement indicated some fraction of the high-temperature phase remaining at 100 K (5%).

### 2.5. Thermal analysis

Differential Scanning Calorimetry (DSC) and Thermal Gravimetric Analysis (TGA) were carried out using the STA 449 F1 Jupiter. Samples (∼4–6 mg) were loaded into aluminium pans with pierced lids and sealed. The temperature programme was as follows: 20 min isothermal step (293 K), heat (293–493 K, 10 K min^−1^), cool (493–293 K, 10 K min^−1^) and a final isothermal step for 5 min (293 K). Helium was used as a purge and protective gas at a rate of 50 ml min^−1^ and 20 ml min^−1^, respectively. Data was analysed using NETZSCH Proteus Thermal Analysis 8.0.2.

### 2.6. FT-IR spectroscopy

From our experiments, the crystallization behaviour of nifedipine from methanol and ethanol is different therefore FT-IR was used to assess the molecular structure in solution to see if there was any difference between the two solutions. The filtered supernatant solution collected after slurrying was analysed prior to crystallization. FT-IR spectra were collected using a Shimadzu IR Spirit Fourier Transform Infrared Spectrophotometer with QATR-S single-reflectance attenuated total reflectance (ATR) probe. Parameters for data collection were transmittance measurement mode and Happ–Genzel apodization. Spectral resolution was set to 4 cm^−1^, with 64 scans per spectrum in the range 400 to 4000 cm^−1^. Background scans of the solvent were conducted between each sample to remove spectra of the pure solvent.

## 3. Results and discussion

Before we discuss the solvate formation in NIF, it is informative to discuss its polymorphism. There are inconsistencies in the nomenclature of NIF in the literature, so to clarify, we will be using the Greek letter notation of the phases. Table 1 ▸ provides a breakdown of the nomenclature in use in the literature and how the notations relate to one another. The α-form (form A) was first solved by Triggle *et al.* (1980 ▸) and is the thermodynamically stable form before melting. The β-, γ- and δ-forms can all be isolated from the melt. The β-form (form C) is produced by crystallization of the liquid state created by heating the amorphous form. The initial determination of the crystal structure was via synchrotron X-ray powder diffraction (Bortolotti *et al.*, 2011 ▸) before subsequent confirmation using single-crystal X-ray diffraction (Gunn *et al.*, 2012 ▸). Two further polymorphs of NIF were discovered by Gui *et al.* (2020 ▸) who varied the crystallization conditions and temperature. The high-temperature β′-form is accessed by heating the β-phase above 333 K, indicating their enantiotropic relationship. The γ′-form is observed by crystallization of the melt at 373 K. Single crystals of this polymorph can be grown by annealing the solid near its melting point (410 K). One of the key observations is that, so far, it can only be observed from isolated liquid droplets and converts readily to the other forms on contact with those crystals (Gui *et al.*, 2020 ▸). The relationship with its parent structure (γ-form) is also enantiotropic and the reversible conversion to the γ-form occurs at 243 K. Finally, the δ-form was crystallized in a two-step process by seeding the melt of NIF with structurally similar compound felodipine then subsequently using the powder of the δ-form to seed droplets of the NIF melt. The use of felodipine enabled NIF to possess the *cis/cis* conformation not seen in the other polymorphs. The unit-cell parameters for each of the known forms are in Table S1. From these observations it would seem likely that the α-, β-, β′- and γ′-forms are likely candidates for the desolvation product.

NIF is a di­hydro­pyridine compound that has shown propensity to form solvate structures. Solvents selected for this study were based on the two NIF solvates deposited in the Cambridge Structural Database (CSD) (Groom *et al.*, 2016 ▸) [1,4-dioxane (N_14DIO_; CSD refcode: ASATOD; Caira *et al.*, 2003 ▸) and di­methyl­sulfoxide (N_DMSO_; CSD refcode: QUPRUP; Klimakow *et al.*, 2010 ▸)]. NIF solvates were produced from various solvent systems: morpholine, tetra­hydro­furan, pyridine, di­methyl­acetamide (two polymorphs), di­methyl­formamide and methanol, in addition to the known 1,4-dioxane and di­methyl­sulfoxide structures. The remaining solvents recrystallized the thermodynamically stable α-NIF from slow evaporation and slurry routes (Fig. S1).

### 3.1. Structural similarity

In the solvent screen component of this study seven new solvated structures were obtained and the rest of this section will describe and discuss these. The new structures can be split into two different groups; (i) those that are structurally similar to the known N_14DIO_ and N_DMSO_ structures, and (ii) those that are significantly different to the previously known solvated forms. One of the key structural observations is that the conformation of nifedipine, in all but the methanol and pyridine solvates, is the same *cis*/*trans* configuration. In the methanol and pyridine structures the acetate groups are orientated in the *cis/cis* configuration, making the molecules symmetrical. The conformational changes alter the interaction with the solvent molecules and the propagation of the crystal structure.

Of the seven new solvated systems, there are three crystals that show structural similarities to the known dioxane and di­methyl­sulfoxide solvated forms. The similarity is centred around the packing of the NIF molecules with the solvent molecules varying the size of the repeating unit. The THF, pyridine and methanol solvates all crystallize in different packing arrangements, but all show intriguing behaviour that will be discussed in the next section.

#### 3.1.1. Dioxane (N_14DIO_) and morpholine (N_MORPH_)

**Figure 1:**
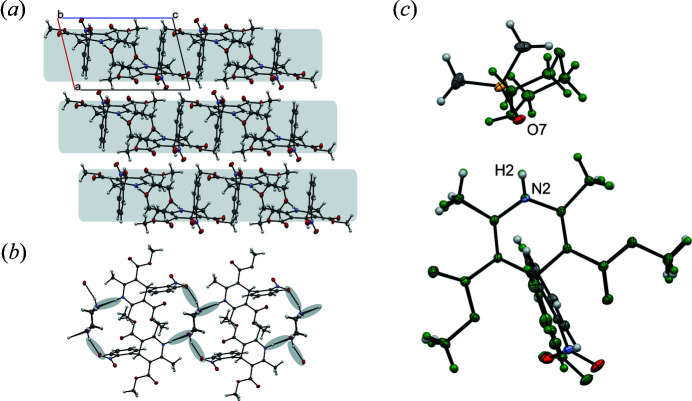
(*a*) Structure of N_14DIO_ shown down crystallographic *b*-axis. Layers are seen in the structure, parallel to the *c*-axis and perpendicular to the *a*-axis highlighted in the figure. Nitro­phenyl groups separate the layers that run perpendicular to the *a*-axis. (*b*) The isostructural morpholine solvate showing both modelled orientations or morpholine, forming a ring structure. (*c*) Overlay of the N_MORPH_ (green) and N_DMSO_ (shown by element) solvates, highlighting the similarity in the position of the solvent molecules in the structure when the main bodies of nifedipine are overlaid.

The crystal structure of the 1,4-dioxane solvate was first described by Caira and co-authors (CSD Refcode: ASATOD; Caira *et al.*, 2003 ▸). The morpholine solvate is isostructural with the dioxane solvate where the asymmetric unit consists of one molecule of NIF and half a molecule of 1,4-dioxane (or morpholine) leading to a 2:1 solvate. In the case of morpholine, the molecule is disordered over the inversion centre with the NH group replacing one of the oxygen atoms. There is no significant change in geometry due to the extra hydrogen bond capability, but the NH group does point towards the nitro group of a neighbouring NIF. The solvent molecule takes a chair conformation and is bonded to the di­hydro­pyridine group of the NIF via a NH⋯O hydrogen bond parallel to the *b*-axis. The main body of the NIF molecule lies approximately on the (210) plane with the nitro­phenyl group lying perpendicular to this (88.59° for N_14DIO_ and 87.62° for N_MORPH_). The solvent molecule in each of the solvates is orientated perpendicular to the NIF backbone [Fig. 1 ▸(*a*)]. In each of the hydrogen-bonded units, the NIF molecules are related by the inversion centre. The hydrogen bonded units form layers in the structure [indicated by the grey rectangles in Fig. 1 ▸(*a*)]. The layers are stacked perpendicular to the *a*-axis with nothing more than van der Waals interactions between the neighbouring layers.

#### 3.1.2. Di­methyl sulfoxide (N_DMSO_)

Despite the obvious differences in the molecular structure of the solvent, the next three structures show significant similarity to the N_14DIO_ and N_MORPH_ structures. The structure of N_DMSO_ was previously determined by Klimakow and co-authors (CSD refcode: QUPRUP; Klimakow *et al.*, 2010 ▸). It crystallizes in 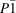 with one NIF and one DMSO molecule in the asymmetric unit. Hydrogen bonding connects the two components between the di­hydro­pyridine nitro­gen of NIF and oxygen of the DMSO (*D*⋯*A*, N2⋯O7) with a distance of 2.8597 (18) Å compared with 2.877 (2) Å observed in the ambient-temperature structure (Klimakow *et al.*, 2010 ▸). Interestingly, the DMSO molecules are arranged in an antiparallel arrangement via the inversion centre which enables the DMSO to take a similar position in the structure as the dioxane molecules leading to the similarity in the structures [Fig. 1 ▸(*c*)] albeit that the unit cell volume is expanded. There are no strong hydrogen-bonding interactions between the layers but the orientation of the nitro­phenyl groups enables weaker CH⋯O interactions to occur with oxygen atoms from the meth­oxy group (O5) and nitro group (O1) of a neighbouring molecule, translated down the *a*-axis.

#### 3.1.3. Di­methyl­acetamide (N_DMA_)

Whilst N_DMSO_ has a unit cell that is broadly similar to that in the N_14DIO_ structure, the di­methyl­acetamide solvate forms a structure that is elongated along the *c*-direction; this can be attributed to the location and orientation of the solvent molecules. N_DMA_ undergoes a phase transition on cooling below 143 K into a low-temperature phase. We have denoted the high-temperature phase α-N_DMA_ and the low-temperature phase β-N_DMA_. To characterize α-N_DMA_, single-crystal diffraction data were collected at 173 K to avoid the phase transition.

**Figure 2:**
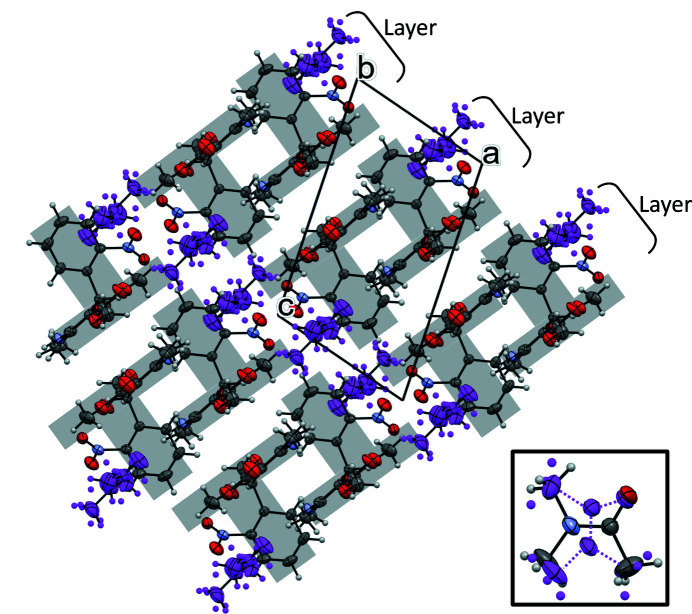
Layers in the N_DMA_ structure. Nitro­phenyl groups are orthogonal to the main body of the nifedipine, fitting in the opposite chain forming a bilayer, forming a T-motif. DMA molecules are shown in purple for visual ease. Inset shows the two orientations DMA occupies in the structure, one shown in purple and the other shown by element.

The hydrogen bonding is consistent with the other solvated forms and via the NIF di­hydro­pyridine group. The main body of the NIF molecules sit on (101) plane with weak CH⋯O interactions with the nitro group between the layers of NIF molecules. The DMA molecule sits almost planar with respect to the di­hydro­pyridine ring of NIF (6.80° between the mean plane of the DMA and di­hydro­pyridine ring) and the other solvent molecules in the channel, with little interaction between them. The DMA molecule shows disorder, occupying two positions in the structure. The shape of DMA enables the solvent molecule to adopt two positions without a significant change in occupied space (Fig. 2 ▸, inset). The methyl groups that are *trans* to one another can ‘swap’ positions whilst maintaining the hydrogen-bonding interaction to the carbonyl oxygen.

**Figure 3:**
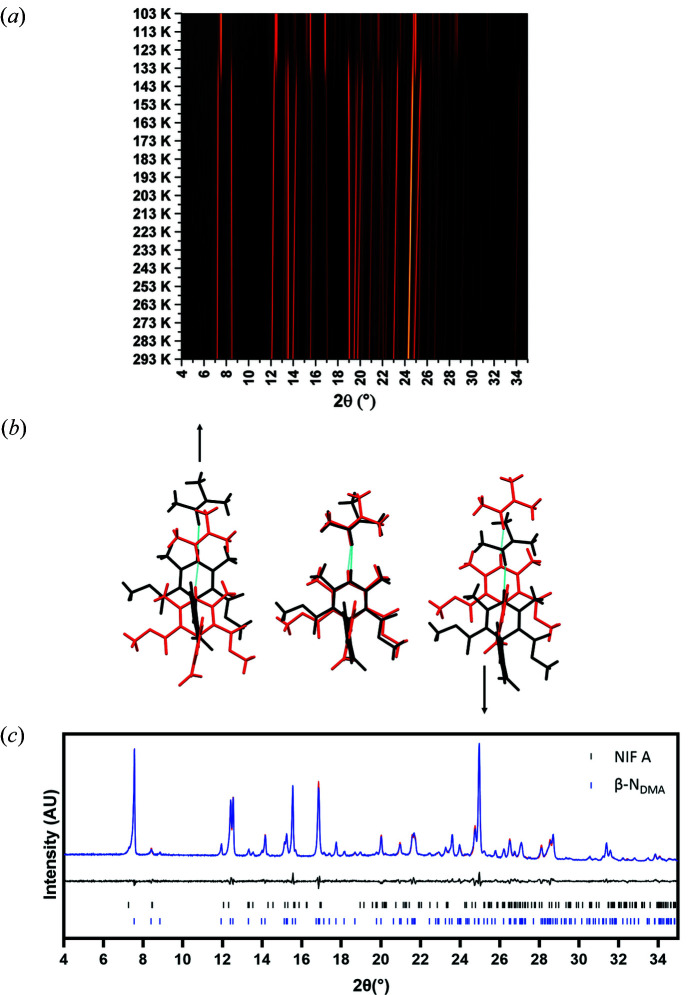
(*a*) The XRPD patterns as a function of cooling, indicating the emergence of the β phase at 143 K; (*b*) the change to the structure from α-N_DMA_ (red) to β-N_DMA_ (black) over the phase transition. The NIF molecules remain in the same plane but move laterally along the direction of the hydrogen bond; (*c*) Rietveld refinement of the pattern collected at 103 K using the structure solution for β-N_DMA_ obtained from powder data (experimental pattern – red, model – blue, difference – black). A minor contribution from NIF A was also identified in pattern, *hkl* tick marks for the component phases are shown as vertical bars (black – NIF A, blue – β-N_DMA_)

Whilst mounting a crystal of N_DMA_ onto the diffractometer with the low temperature device operating at 100 K, the single crystal turned opaque and disintegrated indicating a phase transition had occurred. The reconstructive nature of the transition necessitated the use of powder diffraction to elucidate the structural changes over the transition. A freshly ground sample of N_DMA_ was loaded into a capillary to provide the best quality powder diffraction patterns with minimal chance of the pattern being affected by preferred orientation. Fig. 3 ▸(*a*) shows the surface plot of the XRPD data captured every 10 K from 293 K down to 103 K indicating that a phase transition does occur at 143 K and that β-N_DMA_ remains stable to 103 K. The XPRD pattern reversibly converts back to the α-NIF_DMA_ pattern on return to ambient temperature. The crystal structure of β-N_DMA_ was solved using the X-ray powder diffraction data and found to have a stoichiometric ratio of 1:1 NIF: DMA, maintaining the ratio from α-N_DMA_ but with an ordered solvent molecule. β-N_DMA_ possesses unit-cell parameters that are very close to the original phase with a significant change in the γ angle.

The overall packing of the structure of β-N_DMA_ is significantly different to the α-form with 2 out of 15 molecules identified as being common using the packing similarity methodology in the *Mercury* (Macrae *et al.*, 2020 ▸). The two phases maintain a similar hydrogen-bonding motif but the arrangement of the molecules in the layer is different [Fig. 3 ▸(*b*)]. On cooling to 100 K, neighbouring units of NIF and DMA (approximately along the *b*-axis) move in opposite directions along the hydrogen bond [moving from red arrangement to black arrangement in Fig. 3 ▸(*b*)]. The group to left of the central unit moves upwards whilst the group on the other side moves downwards in the same plane. During this transition, there is a slight conformational change around the nitro­phenyl group of NIF and the DMA molecules no longer lie parallel with the NIF backbone. Whilst the difference of the two structures seems to be relatively small, the lateral movement of all the hydrogen-bonded units is catastrophic for the integrity of the crystal.

#### 3.1.4. Di­methyl­formamide, N_DMF_

N_DMF_ shares structural similarities to α-N_DMA_ with 6 out of 15 molecules overlapping after conducting a structure similarity search in *Mercury* (Macrae *et al.*, 2020 ▸), but we note that there is a difference in collection temperature (100 K versus 143 K). This indicates that just under half of the molecules are located in a similar position between these two phases. At 100 K, β-N_DMA_ shows no overlap with N_DMF_ indicating the large change in the molecular packing between the N_DMA_ forms over the phase transition. In N_DMF_, we observe the same hydrogen-bonding motif [N2⋯O7 = 2.8533 (12) Å]. The DMF molecules reside in a similar location of the structure to the DMA but the DMF molecules sit more perpendicular with respect to the main body of the NIF molecules (65.49°). The solvent molecules are fully ordered (unlike the high-temperature phase), sitting in channels parallel to the *a*-axis. In this orientation, the DMF can interact with the meth­oxy group of the NIF molecules in the layer above and a second symmetry-related molecule interacts from above.

### 3.2. Thermal analysis of structurally similar compounds

The focus of the paper was to determine the desolvation process in NIF solvates to observe if the metastable forms could be accessed through this route. To elucidate the changes on desolvation we used both differential scanning calorimetry and thermogravimetric analysis of the powders in combination with variable-temperature powder X-ray diffraction measurements. For all the structurally similar systems under study, the crystal structures show that on heating the expansion is anisotropic, with greater increase in the *a*-axis which is perpendicular to the layers in the structure where hydrogen bonding is absent. The desolvation for N_14DIO_ and N_MORPH_ is straightforward and clean with a clear transition from the solvate to the α-form of NIF beginning at 373 K and is complete by 408 K (for N_14DIO_, Fig. S2) whilst N_MORPH_ desolvates a little earlier at 358 K with completion by 388 K (Fig. S3). The temperature is consistent with the observations of the desolvation of N_DIO_ by Caira *et al.* (2003 ▸). There is a small loss of morpholine at 320 K that may suggest some residual surface solvent escaping at this temperature. The solvent molecules are located in channels parallel to the *b*-axis, so it is likely that on thermal expansion, solvent molecules can easily escape the structure. Over the timescale of the XRPD run there is no indication of amorphization, hence the transition to the thermodynamically stable α-form is via the rearrangement of the crystalline phases.

N_DMSO_ and N_DMA_ show slightly different behaviour to the solvates previously described, where both show a significant decrease in the crystallinity of the solid as we approach the melting point of NIF. N_DMSO_ shows a constant mass loss from 293 K towards the maximum temperature reached, 473 K, indicating that there may be some decomposition occurring in solution (Fig. S4). The DSC trace of N_DMSO_ is rather unusual and does not look as clean as N_14DIO_ and N_MORPH_. There is a prolonged endothermic event beginning at 310 K and complete by 350 K that cannot easily be ascribed to any event. VT-XRPD gives us a clearer indication of the change to the crystal structure as a function of the desolvation and indicates that the solvate undergoes a transition to α-NIF at 363 K; the difference in the temperature (compared to DSC) can be attributed to the different sample environments, ramp rates and holding temperatures for the collection of the XRPD data. Consistent with N_14DIO_ and N_MORPH_, the DMSO molecules are located within channels in the crystal structure which should aid the escape of the solvent (Liu *et al.*, 2019 ▸). In this solvate, we observed that the diffraction patterns near the melt temperature are of poor quality. There is a general reduction in reflection intensity and observation of an amorphous background, which together suggests the melting of the solvate; this is indicated by the increase in the background colour in Fig. S4. The lower crystallinity after the melting event and appearance of α-NIF suggests that the DMSO released from the structure stays in the capillary and coalesces as a liquid for the NIF to dissolve and recrystallize. This idea is supported by the DSC trace, where there is a very broad endotherm of NIF rather than the sharp event associated with a melt. N_DMA_ shows complete desolvation by 383 K to α-NIF. The mass loss of 23.61% is equivalent to the mass of DMA indicating that it is a complete process (Fig. S5). In a similar manner to N_DMSO_, there is the appearance of an amorphous structure in the background on desolvation that indicates a similar process where DMA acts as the solvent to dissolve NIF.

**Figure 4:**
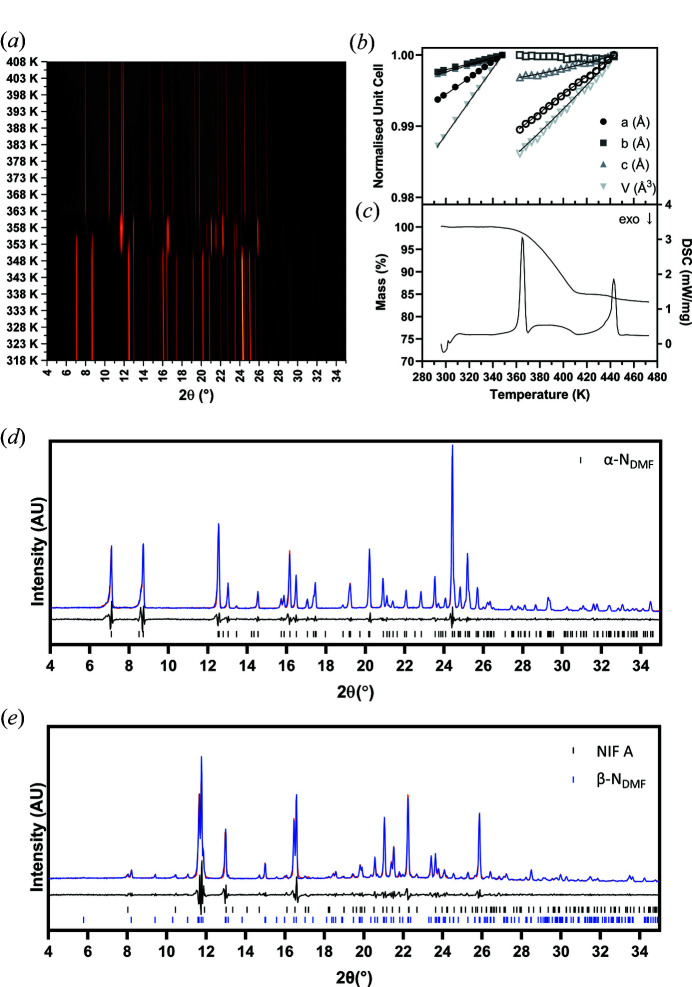
Thermal data of N_DMF_ capturing the desolvation to α-NIF. (*a*) Surface plot of VT-XRPD data from 318 K to 408 K. (*b*) Unit-cell parameters from Rietveld refinements of each XRPD pattern. Closed symbols represent data for the solvate; open symbols represent data for desolvated structure. The data were fitted with the Berman equation of state (Angel *et al.*, 2014 ▸; Gonzalez-Platas *et al.*, 2016 ▸; Berman, 1988 ▸). (*c*) DSC and TGA traces for N_DMF_. (*d*) XRPD pattern for the DMF solvate collected at 293 K [the model (blue) is superimposable on the data (red) indicating a good fit]. (*e*) XRPD pattern obtained at 358 K (after phase change). The pattern is well accounted for by indexed phase (β-N_DMF_) and minor component of NIF A. For both fitted patterns [(*d*) and (*e*)], the experimental data are shown in blue, whilst the calculated profile is shown in red. The difference profile is displayed underneath the diffraction pattern.

N_DMF_ shows interesting behaviour on heating that the other materials have failed to exhibit thus far. The expansion of the unit-cell parameters for the solvate can be fitted up to 348 K, and beyond 363 K they can be fit to α-NIF. Between these temperatures, however, reflections for another form are observed in the VT-XRPD (Fig. 4 ▸). These reflections cannot be attributed to any of the metastable forms of NIF (Fig. S6). From the VT data, the X-ray pattern at 358 K provided the best opportunity to solve the new phase. This pattern displays some diffraction that can be attributed to α-NIF but this is present at a low level. Using *Topas* we were able to identify those diffraction intensities that were attributable to both α-NIF and N_DMF_ and discount these from our list of reflections for indexing (the latter was not present). The other unindexed peaks were identified and used to index the unknown phase. After numerous attempts in *DASH* (David *et al.*, 2006 ▸), *Topas* (Coelho, 2005 ▸) and *N-TREOR* (Altomare *et al.*, 2000 ▸) a consistent unit cell was identified [*a* = 15.436400 Å, *b* = 15.20191 Å, *c* = 9.50456 Å, 90°, 98.3578°, 90°] in space group *P*2_1_. The Pawley fit to the data using this cell is good with *R*_wp_ = 10.17% for the mixed phase [Fig. 4 ▸(*e*)]. The unit-cell volume of 2205 Å^3^ indicates that the structure contains two molecules of NIF and two molecules of DMF based on the 18 Å^3^ rule (*i.e.* each non-H atom has a volume of 18 Å^3^). Attempts were made to solve the structure in *DASH* (David *et al.*, 2006 ▸), *FOX* (Favre-Nicolin & Černý, 2002 ▸) and *EXPO* (Altomare *et al.*, 2013 ▸) using the space groups *P*2, *P*2_1_ and *P*2_1_/*m* but no satisfactory solution was found albeit that the χ^2^ values were promising. The lack of structural solution for this additional phase (designated β-N_DMF_) is frustrating but from the analysis that we have performed it appears that the phase transition is a change in polymorph rather than a loss of solvent. The thermal identification of this transition is complicated by the fact that the transition is close in temperature to the desolvation temperature hence is masked in the thermal analysis (assuming the conversion occurs in the DSC/TGA under slightly different heating parameters). The TGA indicates there are two different weight loss events the first of which is a continuous weight loss of 15.01% before a second discrete loss of 1.97% released on melting [Fig. 4 ▸(*c*) and Table 2 ▸]. The calculated weight loss for the desolvation for a 1:1 NIF:DMF solvate is 17.43% which confirms the total loss during the experiment (16.98%).

### 3.3. Novel solvate structures

#### 3.3.1. Tetra­hydro­furan (N_THF_)

**Figure 5:**
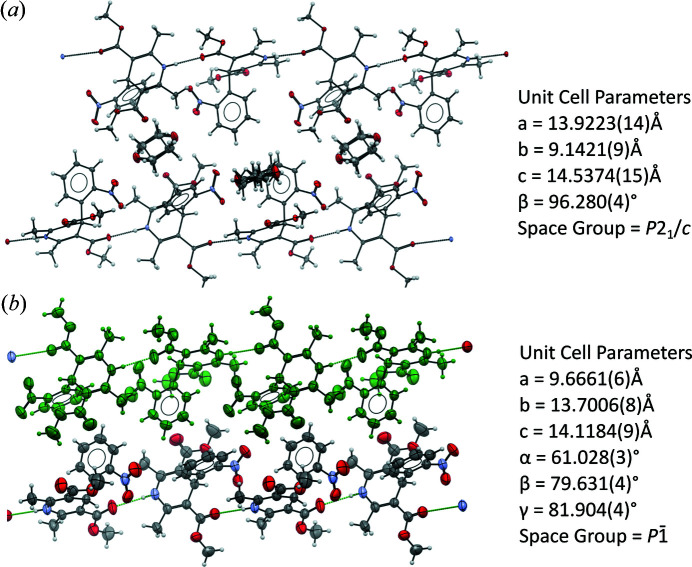
The similarities in the hydrogen-bonding chains in (*a*) N_THF_ and (*b*) β-form of NIF indicating the similarity. Neighbouring chains are translated to accommodate the THF molecules in the structure. The green colouring of β-form to highlight the neighbouring chain. Both structures were collected at 100 K. The unit-cell parameters for the β-form have been taken from the CSD (refcode: BICCIZ02; Gunn *et al.*, 2012 ▸) and transformed to correspond to the N_THF_ solvate which shows the commonality in the unit-cell lengths (Groom *et al.*, 2016 ▸).

Tetra­hydro­furan (THF), a cyclic ether, can form one hydrogen bond via its oxygen atom. Although collected at 100 K, the modelled structure showed disorder, with the solvent molecules sitting on an inversion centre despite not being centrosymmetric which would open up the potential for hydrogen bonding in opposite directions (Fig. 5 ▸). Despite this, the THF is not involved in any hydrogen-bonding interactions at all. NIF molecules form hydrogen bonds between the nitro­gen in the di­hydro­pyridine (N2) and the ester carbonyl group of a neighbouring molecule (O6) that resembles the metastable polymorph of NIF, β-form (Fig. 5 ▸). The unit-cell parameters and the hydrogen bonding in this structure are similar to β-NIF but the NIF molecules are symmetry-equivalent in N_THF_. The differences between N_THF_ and the β-form of NIF show that there is a translational change in the neighbouring chains that allows the THF to sit in pockets between the chains made by the nitro­phenyl rings. There are no opportunities in this pocket for the THF molecules to engage in hydrogen bonding. The addition of the THF into the structure has altered the O2—N1—C1—C2 dihedral angle from 143.29° and 151.77° in molecules 1 and 2 of the β-form to 133.20 (9)° in the N_THF_ structure.

In N_THF_, the expansion of the structure is greatest in the direction of the *a*- and *b*-axes where there is no hydrogen bonding present. These axes exhibit very similar behaviour (Fig. S7). The DSC indicates that desolvation takes place between 349 and 361 K, above the boiling point of THF at 339 K, with a single desolvation event. There is some discrepancy between the DSC and VT-XRPD where the diffraction indicates that the desolvation starts to take place as early as 313 K. This can be attributed to the differences in the experimental process, *e.g.* grinding the powder before XRPD analysis and sample holder, *i.e.* aluminium pan versus boro­silicate capillary. The process of grinding the sample is likely to have the greatest impact, imparting energy into the material and increasing the surface area for quicker desolvation. From the Rietveld analysis of the data collected at 323 K, additional peaks of the α-form of NIF were observed. N_THF_ is present in detectable quantities until 348 K where the conversion is complete; the unit-cell parameters can only be fitted up to 338 K due to the low level of the N_THF_ present in the sample beyond this temperature. From the Bravais–Friedel–Donnay–Harker morphology prediction of the crystal shape, the THF molecules reside in layers on the largest face of the crystal surfaces that would facilitate the desolvation (Fig. S7). Although N_THF_ and β-form NIF are structurally similar, we do not see reflections corresponding to β-form during the desolvation. This is somewhat surprising given the structure of the solid. However, Grooff *et al.* (2007 ▸) observed that the β-form easily converts to the thermo­dynamically stable α-form of NIF via the metastable β′-form at approximately 343 K on heating which is consistent with our observations. TGA data can also be used to confirm the stoichiometry of the crystal structure that is provided by single-crystal XRD. The calculated ratio of NIF to THF is 2:1, with a weight loss of 9.43% after desolvation, which agrees with the experimental data (9.48%).

#### 3.3.2. Pyridine and methanol

**Figure 6:**
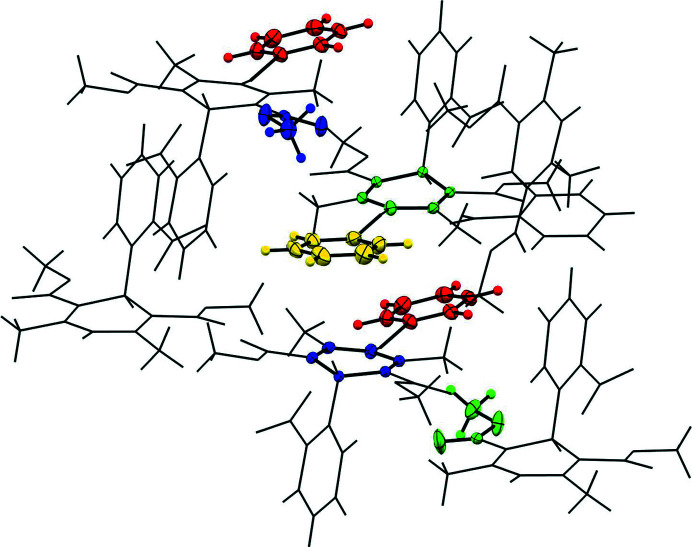
Structure of N_PYRI_ indicating the antiparallel stacking of the pyridine molecules with the di­hydro­pyridine ring system of NIF. The pyridine molecules are sandwiched between di­hydro­pyridine ring and meth­oxy group of a neighbouring molecule. Colour scheme is by symmetry equivalence.

**Pyridine (N_PYRI_)**. The asymmetric unit of N_PYRI_ contains two NIF and two pyridine molecules. This solvate crystallizes in *P*2_1_ with unit-cell lengths of 9.4363 (10) Å, 14.4247 (14) Å and 15.1387 (15) Å for *a*, *b* and *c*, respectively, and a β angle of 96.160 (3)°. NIF interacts through the di­hydro­pyridine and the pyridine nitro­gen with the main body of the NIF molecule lying parallel to the pyridine (red and blue, yellow and green). These symmetry inequivalent pairs stack in an antiparallel manner at a distance just beyond the van der Waals radii of the atoms (Fig. 6 ▸). The pyridine molecules are located in small pockets, sandwiched between the meth­oxy group of a molecule translated along the *a*-axis and ring system of the second NIF molecule in the asymmetric unit. As we extend out along *c*-axis, the close contacts between the nitro groups and neighbouring meth­oxy group become evident.

Interestingly, the diffraction patterns produced for pyridine via the slurry method showed the sample to be α-NIF indicating that the pyridine solvate is likely to be the metastable form. Therefore, we needed to change our method to produce the powder for desolvation analysis. Single crystals produced by slow evaporation were ground into the powder which may have affected the desolvation process through the extra manipulation of the solid during the preparation of the powder. During VT-XRPD studies, N_PYRI_ shows noticeable expansion in the *b*-axis, the direction perpendicular to the layers within the crystal structure, where there is only π–π interactions between molecules. The desolvation of the sample is rapid, with full conversion to the α-form of NIF by 323 K. Unit-cell parameters could only be determined between 293 K and 313 K but with the limited data, it suggests that the expansion is anisotropic. DSC for N_PYRI_ shows desolvation of the system has an onset of ∼304 K and is complete by ∼352 K, below the boiling point of pyridine (388 K) (Fig. S8). The DSC graph shows a structured endotherm, with overlapping peaks observed during desolvation which can be attributed to the different symmetry-inequivalent pyridine molecules being released at slightly different times. The TGA indicates that the release is continuous with some variation in the rate as opposed to the distinct two-step desolvation observed in furosemide-THF and furosemide-DMF samples (Minkov *et al.*, 2014 ▸).

**Figure 7:**
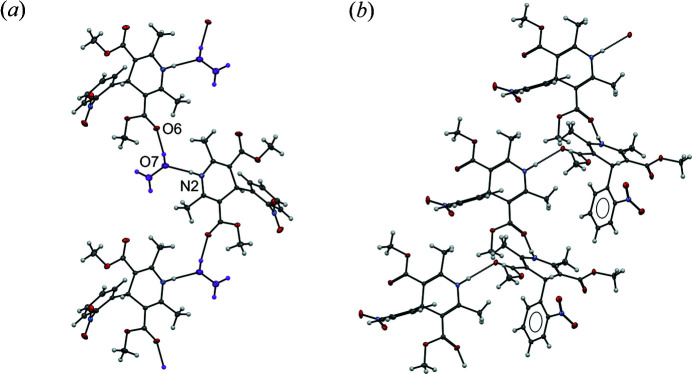
The structure of (*a*) N_MeOH_ and (*b*) α-NIF showing the similarities in structure between the two forms. The loss of methanol from the structure would enable the NH⋯O interaction to form without significant change in the packing.

**Methanol (N_MeOH_)**. The final structure is N_MeOH_ which was an unexpected solvate isolated from the slow evaporation from a methano­lic solution. A slurry of NIF in methanol resulted in crystals of the α-form of NIF similar to the N_PYRI_. This seems to be a unique observation of an alcohol system. Previous literature and our own studies did not observe an ethanol or 2-propanol solvate and both crystallized α-NIF (Li *et al.*, 2020 ▸). N_MeOH_ is dissimilar to the solvates reported previously; methanol forms two hydrogen bonds via the hydroxyl group, acting as both donor and acceptor to two NIF molecules [N2⋯O7 = 2.9201 (11) Å and O7⋯O6 = 2.7995 (10) Å]. The ability of the methanol to form two hydrogen bonds leads to a chain structure parallel to the *b*-axis. This hydrogen-bonded chain structure resembles α-NIF, which has hydrogen bonds between a carbonyl group and pyridine of neighbouring molecule (Fig. 7 ▸). The main body of the NIF is parallel to the 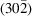 plane and the chains above and below are linked through an inversion centre with little interaction between layers other than van der Waals interactions. There is little interaction between neighbouring supramolecular columns along the *a*-axis.

**Figure 8:**
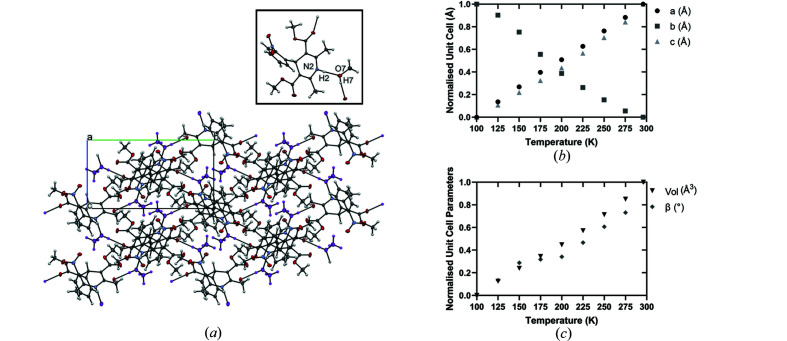
(*a*) N_MeOH_ viewed down the *a*-axis with methanol molecules shown in purple residing in the channels parallel to *a*-axis. The inset shows the atoms involved in the hydrogen bonding between methanol and nifedipine. Nonlinear thermal behaviour of N_MeOH_ showing the (*b*) decrease in *b*-axis and (*c*) increase in β angle and volume with increasing temperature.

Similar to N_PYRI_, the slurry of NIF in methanol did not produce the solvated form, therefore single crystals produced from the slow evaporation from the saturated solution were pulverized for analysis. Interestingly, N_MeOH_ displays negative thermal expansion behaviour along its *b*-axis (Fig. S9). Negative thermal expansion (NTE) is a mechanism predominantly reported for metal–organic frameworks (Goodwin & Kepert, 2005 ▸; Wu *et al.*, 2008 ▸; Lock *et al.*, 2010 ▸). Authors describe the mechanism as having a wine-rack expansion; with expansion in one direction causing contraction in a second direction (Cliffe & Goodwin, 2012 ▸). A recent publication by van der Lee and Dumitrescu explains how NTE is common amongst organic crystals, but rarely reported (van der Lee & Dumitrescu, 2021 ▸). In N_MeOH_, chains of NIF and solvent molecules align in the direction of the *b*-axis, with the hydrogen bonds between methanol and carbonyl group of the NIF (O7H7⋯O6). When viewed down the crystallographic *a*-axis, the methanol molecules are easily distinguished indicating channels throughout the crystal structure (Fig. 8 ▸, purple molecules). A possible mechanism of solvent loss is that as temperature increases, the channel structure enables the escape of solvent molecules. The loss of methanol, and its bridging interaction, causes the contraction of the NIF molecules together bridging the gap to form the hydrogen bond between the di­hydro­pyridine and the carbonyl of the ester (observed in α-NIF), which causes the contraction in the *b*-axis (Fig. 8 ▸). STA indicates that the onset of desolvation took place prior to analysis; TGA shows a mass loss of 1.69% prior to the melt of α-NIF, rather that the ideal value for the solvate of 8.47% using the stoichiometry determined from the crystal structure. This indicates that the solvent is lost easily and as soon as the crystal is taken out of the solvent.

Further investigation into the negative thermal expansion was conducted using SC-XRD data at low temperature as a result of the small window of opportunity at higher temperatures due to desolvation. A single crystal was collected at 100 K with subsequent collections increasing by 25 K [Figs. 8 ▸(*b*) and 8 ▸(*c*)] and indicate that the NTE behaviour extends from 100 K to the desolvation temperature of 340 K. As the temperature is increased there is a reduction in the *b*-axis but expansion along the *c*-axis that can clearly be observed in the animated GIF (in the supporting information) at 25 K intervals. There is a slight rotation of the body of the NIF that appears to allow the narrowing in along the *b*-direction. The fact that this still occurs at such low temperatures indicates that the NTE is not associated with the loss of solvent on heating but from a rearrangement of the molecules on warming the sample.

### 3.4. Solution study – ethanol and methanol

**Figure 9:**
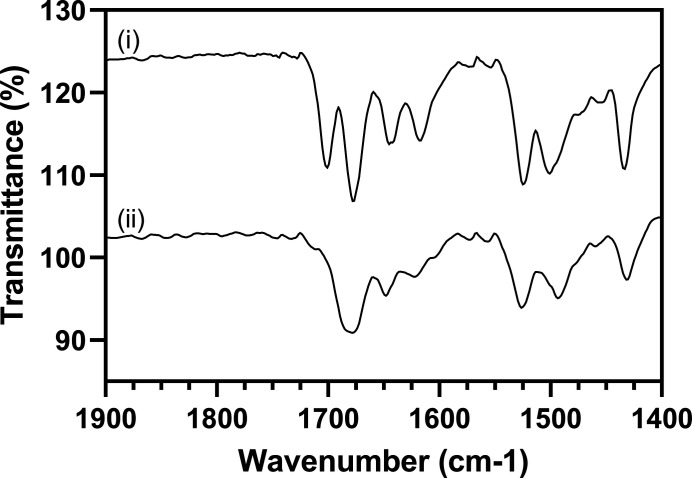
FTIR spectra of NIF in (i) methanol and (ii) ethanol, between 1400 and 1900 cm^−1^, highlighting the region where we see the carbonyl stretch. The background of the respective solvents was removed to aid in the identification of NIF.

Despite the successful isolation of a methanol solvate we did not observe a solvate with ethanol. There are a number of reasons behind the difference in the outcomes of the crystallization. Firstly, the two solvents involved show a variation in rate of evaporation which can affect their ability to be incorporated into the crystal structure. Secondly, the solution structure of the solvent molecules may include clusters of solvent molecules that prohibit the incorporation into the crystal structure. To investigate this scenario further, saturated solutions of NIF in the two solvents were analysed using FTIR. The solubility of NIF in methanol and ethanol at 20°C are 26 mg ml^−1^ and 17 mg ml^−1^, respectively (Ali, 1990 ▸). Fig. 9 ▸ shows the spectra of the filtered saturated solutions, with the background of the respective solvent removed. The IR spectrum of NIF was first discussed by Burger & Koller (1996 ▸) and is the basis of our assignment of vibrations in this study. The carbonyl region of the spectra is where differences between NIF in the methanol and ethanol solutions are observed. The IR spectra indicate that the solvent interaction with NIF is causing a change to the carbonyl stretch of the meth­oxy groups in NIF (regions between 1650–1730 cm^−1^ and 1670–1725 cm^−1^ for methanol and ethanol, respectively). The shift to lower wavenumber of the ester stretch can be attributed to the connectivity to the unsaturated bonds in the di­hydro­pyridine ring enabling resonance stabilization hence a weakening of the stretch. The stretching frequency of the ester group in the ethanol solution is a broader single peak indicating that it is likely composed of two bands with slight differences in energy of the carbonyl group, *i.e.* there is little difference in the intermolecular interactions of each carbonyl group. In methanol, the stretching frequencies of the carbonyl groups significantly changes indicating that the environment around each carbonyl is different. This correlates very well with the observation in the solid-state where the methanol is hydrogen bonded to one of the ester groups whilst the second ester group is only involved in van der Waals contacts with neighbouring CH groups from the nitro­phenyl substituent (Fig. S10*h* ). The change in the vibrational behaviour indicates that the interactions could be preformed in the solution phase before they are crystallized (Du *et al.*, 2015 ▸). This may account for the difference in the outcomes from the slurry and solvent evaporation routes; the latter providing the opportunity for the interactions to form without the more stable α-NIF being present.

### 3.5. Rationale for solvate formation

The solvents chosen were based on the solvates that had already been identified in the literature (DMSO and dioxane). We believe that the reason for their solvate behaviour is based on several factors. Firstly, for the most part, the solvents only possess hydrogen-bond donors that can receive the hydrogen bond from nifedipine, with the solvents used interacting through a hydrogen bond via the amine moiety. Secondly, the size of the solvent molecules also seems to be a feature of these solvates. The molecules that have successfully formed a solvate have a diameter between 5 and 6 Å at their widest point apart from that with the methanol solvate whose diameter is closer to 3 Å. This is important as the dynamic behaviour of molecules during nucleation will be a factor in the successful formation of the crystal. Finally, the packing of the molecules into the crystal structure may be a factor. For those molecules that fit the size range but do not form solvates, *e.g.* ethanol and 2-propanol, the insertion of these molecules into the methanol structure may cause the structure to become energetically unfavourable. The accepting and donating capability of the alcohol requires a different packing arrangement of nifedipine molecules based on the methanol solvate crystal structure. In this structure, there is no significant space available in the environment around the methyl group of the methanol to enable the insertion of ethanol or propanol without a significant rearrangement of the molecular packing.

## 4. Conclusion

A solvent screen of NIF has identified seven new solvates, adding to the structural landscape of this pharmaceutical material. The series of solvate structures can be divided into those that are structurally similar to the known dioxane and DMSO solvates (morpholine, DMA and DMF) and those that have novel structures (THF, pyridine and methanol). Most of the solvates interacted with the solvent through the di­hydro­pyridine hydrogen-bond donor except for the N_THF_ structure, which possessed a hydrogen-bonding pattern that was similar to the metastable β-NIF. In each case, the hydrogen bonding in the solvates did not substantially affect the outcome of the desolvation process; desolvation gave the known thermodynamically stable α-NIF. We have identified that N_DMA_ and N_DMF_ possess enantiotropically related polymorphs with a reconstructive phase transition occurring between α-N_DMA_ to the low-temperature phase β-N_DMA_ at 143 K on cooling, and the transition occurring in N_DMF_ at ∼353 K on heating. β-N_DMA_ was solved using the powder diffraction data and shows that the disorder that is present in α-N_DMA_ is resolved on cooling the system; the higher temperature N_DMF_ polymorph remains unsolved but our analysis, so far, suggests it is a polymorph of the 1:1 solvate. One of the significant findings is that a methanol solvate can be isolated, but an ethanol solvate was not observed. The behaviour in solution indicates that the interaction with methanol molecules is preformed in the solution phase before the crystallization occurs. A rationale for the lack of ethanol solvate could be attributed to the extra methyl group in the solvent that affects the ability to pack into an energetically competitive crystal structure. This study has increased our knowledge of the solid-state landscape of nifedipine but also provided structural information on desolvation routes of these novel phases. This additional information can be vital in the search for novel pathways to polymorphism in pharmaceutical materials.

## Supplementary Material

Crystal structure: contains datablock(s) nif_14dio_100k, nif_dma_173k, nif_dmf_100k, nif_dmso_100k, nif_meoh_100k, nif_morph_100k, nif_pyri_100k, nif_thf_100k, Beta-NDMA_1, 22_NIF_DMA_100K. DOI: 10.1107/S2052520623001282/aw5077sup1.cif

Structure factors: contains datablock(s) I. DOI: 10.1107/S2052520623001282/aw5077Isup2.hkl

Structure factors: contains datablock(s) nif_14dio_100k. DOI: 10.1107/S2052520623001282/aw5077nif_14dio_100ksup3.hkl

Structure factors: contains datablock(s) nif_dma_173k. DOI: 10.1107/S2052520623001282/aw5077nif_dma_173ksup4.hkl

Structure factors: contains datablock(s) nif_dmf_100k. DOI: 10.1107/S2052520623001282/aw5077nif_dmf_100ksup5.hkl

Structure factors: contains datablock(s) nif_dmso_100k. DOI: 10.1107/S2052520623001282/aw5077nif_dmso_100ksup6.hkl

Structure factors: contains datablock(s) nif_meoh_100k. DOI: 10.1107/S2052520623001282/aw5077nif_meoh_100ksup7.hkl

Structure factors: contains datablock(s) nif_morph_100k. DOI: 10.1107/S2052520623001282/aw5077nif_morph_100ksup8.hkl

Structure factors: contains datablock(s) nif_pyri_100k. DOI: 10.1107/S2052520623001282/aw5077nif_pyri_100ksup9.hkl

Structure factors: contains datablock(s) nif_thf_100k. DOI: 10.1107/S2052520623001282/aw5077nif_thf_100ksup10.hkl

Rietveld powder data: contains datablock(s) Beta-NDMA_1, 22_NIF_DMA_100K. DOI: 10.1107/S2052520623001282/aw5077Beta-NDMA_1sup11.rtv

Supporting information file. DOI: 10.1107/S2052520623001282/aw5077sup12.pdf

MeOH expansion. DOI: 10.1107/S2052520623001282/aw5077sup13.gif

CCDC references: 2173474, 2173475, 2173476, 2173477, 2173478, 2173479, 2173480, 2173481, 2173482

## Figures and Tables

**Table 1:** Nomenclature of the nifedipine polymorphs and their relationship between the different literature sources as well as the two known solvates of nifedipine

Burger & Koller (1996 ▸)	Grooff *et al.* (2007 ▸, 2011 ▸)	Gunn *et al.* (2012 ▸)	Gui *et al.* (2020 ▸)
(I)	Form (A)	α	α
(II)	Form (B)	β′	β′
(III)			γ′ then γ @ 247 K
	Form (C)	β	β
		X†	
			δ
Solvates of nifedipine			
Klimakow *et al.* (2010 ▸)	Caira *et al.* (2003 ▸)		
DMSO	Dioxane		

† Identified through powder diffraction but no further characterization has been done nor phase observed in studies to date.

**Table 2:** Thermal analysis data for NIF solvates

Solvate	Expected mass loss (%)†	Observed mass loss (%)‡	Onset temperature of desolvation (K)	Enthalpy of desolvation (kJ mol^−1^)	Boiling point of solvent (K)
N_14DIO_	11.28	8.07	392.1	19.28	374
N_MORPH_	11.17	11.81	378.1	19.58	402
N_THF_	9.43	9.48	349.2	21.41	339
N_PYR_	18.59	17.06	325.8	35.40	388
N_DMSO_	18.41	32.47	320.9	23.00	462
N_DMA_	20.10	23.61	369.3	57.39	438
N_DMF_	17.43	16.98	362.2	30.85	426
N_MeOH_	8.47	1.69	328.9	9.78	338

† Calculated from SC-XRD data.

‡ Calculated from TGA data.

## References

[bb1] Aitipamula, S., Chow, P. S. & Tan, R. B. H. (2011). *CrystEngComm*, **13**, 1037–1045.

[bb2] Ali, S. L. (1990). *Analytical Profiles of Drug Substances*, Vol. 18, edited by K. Florey, A. A. Al-Badr, G. A. Forcier, H. G. Brittain & L. T. Grady, pp. 221–288. Academic Press.

[bb3] Altomare, A., Cuocci, C., Giacovazzo, C., Moliterni, A., Rizzi, R., Corriero, N. & Falcicchio, A. (2013). *J. Appl. Cryst.* **46**, 1231–1235.

[bb4] Altomare, A., Giacovazzo, C., Guagliardi, A., Moliterni, A. G. G., Rizzi, R. & Werner, P.-E. (2000). *J. Appl. Cryst.* **33**, 1180–1186.

[bb5] Angel, R., Alvaro, M. & Gonzalez-Platas, J. (2014). *Z. Kristallogr. Cryst. Mater.* **229**, 405–419.

[bb6] Berman, R. G. (1988). *J. Petrol.* **29**, 445–522.

[bb7] Bhardwaj, R. M., Price, L. S., Price, S. L., Reutzel-Edens, S. M., Miller, G. J., Oswald, I. D. H., Johnston, B. F. & Florence, A. J. (2013). *Cryst. Growth Des.* **13**, 1602–1617.

[bb8] Bodart, L., Prinzo, M., Derlet, A., Tumanov, N. & Wouters, J. (2021). *CrystEngComm*, **23**, 185–201.

[bb9] Bortolotti, M., Lonardelli, I. & Pepponi, G. (2011). *Acta Cryst.* B**67**, 357–364. 10.1107/S0108768111021653 21775814

[bb10] Boultif, A. & Louër, D. (2004). *J. Appl. Cryst.* **37**, 724–731.

[bb11] Braun, D. E., Bhardwaj, R. M., Florence, A. J., Tocher, D. A. & Price, S. L. (2013). *Cryst. Growth Des.* **13**, 19–23. 10.1021/cg301506x PMC3557919 23378823

[bb12] Braun, D. E., Gelbrich, T., Kahlenberg, V., Tessadri, R., Wieser, J. & Griesser, U. J. (2009). *Cryst. Growth Des.* **9**, 1054–1065. 10.1021/acs.cgd.0c00777 PMC7472434 32913424

[bb13] Bruker (2012). *SAINT*, version 8.40B. Bruker AXS Inc., Madison, Wisconsin, USA.

[bb14] Burger, A. & Koller, K. T. (1996). *Sci. Pharm.* **64**, 293–301.

[bb15] Caira, M. R., Robbertse, Y., Bergh, J. J., Song, M. & De Villiers, M. M. (2003). *J. Pharm. Sci.* **92**, 2519–2533. 10.1002/jps.10506 14603498

[bb16] Cliffe, M. J. & Goodwin, A. L. (2012). *J. Appl. Cryst.* **45**, 1321–1329.

[bb17] Coelho, A. A. (2005). *J. Appl. Cryst.* **38**, 455–461.

[bb18] Coelho, A. A. (2018). *J. Appl. Cryst.* **51**, 210–218.

[bb19] Cosier, J. & Glazer, A. M. (1986). *J. Appl. Cryst.* **19**, 105–107.

[bb20] David, W. I. F., Shankland, K., van de Streek, J., Pidcock, E., Motherwell, W. D. S. & Cole, J. C. (2006). *J. Appl. Cryst.* **39**, 910–915.

[bb21] Dolomanov, O. V., Bourhis, L. J., Gildea, R. J., Howard, J. A. K. & Puschmann, H. (2009). *J. Appl. Cryst.* **42**, 339–341.

[bb22] Du, W., Cruz-Cabeza, A. J., Woutersen, S., Davey, R. J. & Yin, Q. (2015). *Chem. Sci.* **6**, 3515–3524. 10.1039/c5sc00522a PMC5814770 29511513

[bb23] Favre-Nicolin, V. & Černý, R. (2002). *J. Appl. Cryst.* **35**, 734–743.

[bb24] Furuta, H., Mori, S., Yoshihashi, Y., Yonemochi, E., Uekusa, H., Sugano, K. & Terada, K. (2015). *J. Pharm. Biomed. Anal.* **111**, 44–50. 10.1016/j.jpba.2015.03.008 25854856

[bb25] Gonzalez-Platas, J., Alvaro, M., Nestola, F. & Angel, R. (2016). *J. Appl. Cryst.* **49**, 1377–1382.

[bb26] Goodwin, A. L. & Kepert, C. J. (2005). *Phys. Rev. B*, **71**, 140301.

[bb27] Grooff, D., De Villiers, M. M. & Liebenberg, W. (2007). *Thermochim. Acta*, **454**, 33–42.

[bb28] Grooff, D., Liebenberg, W. & De Villiers, M. M. (2011). *J. Pharm. Sci.* **100**, 1944–1957. 10.1002/jps.22419 21259235

[bb29] Groom, C. R., Bruno, I. J., Lightfoot, M. P. & Ward, S. C. (2016). *Acta Cryst.* B**72**, 171–179. 10.1107/S2052520616003954 PMC4822653 27048719

[bb30] Gui, Y., Yao, X., Guzei, I. A., Aristov, M. M., Yu, J. & Yu, L. (2020). *Chem. Mater.* **32**, 7754–7765.

[bb31] Gunn, E., Guzei, I. A., Cai, T. & Yu, L. (2012). *Cryst. Growth Des.* **12**, 2037–2043.

[bb32] Kaur, N. & Suryanarayanan, R. (2021). *J. Pharm. Sci.* **110**, 3743–3756. 10.1016/j.xphs.2021.08.006 34384799

[bb33] Klimakow, M., Rademann, K. & Emmerling, F. (2010). *Cryst. Growth Des.* **10**, 2693–2698.

[bb34] Lee, A. van der & Dumitrescu, D. G. (2021). *Chem. Sci.* **12**, 8537–8547. 10.1039/d1sc01076j PMC8221191 34221335

[bb35] Li, W., Shi, P., Du, S., Wang, L., Han, D., Zhou, L., Tang, W. & Gong, J. (2020). *J. Cryst. Growth*, **552**, 125941.

[bb36] Liu, X., Tang, C. C., Boldyreva, E. V. & Pulham, C. R. (2019). *Cryst. Growth Des.* **19**, 7315–7323.

[bb37] Lock, N., Wu, Y., Christensen, M., Cameron, L. J., Peterson, V. K., Bridgeman, A. J., Kepert, C. J. & Iversen, B. B. (2010). *J. Phys. Chem. C*, **114**, 16181–16186.

[bb38] Macrae, C. F., Sovago, I., Cottrell, S. J., Galek, P. T. A., McCabe, P., Pidcock, E., Platings, M., Shields, G. P., Stevens, J. S., Towler, M. & Wood, P. A. (2020). *J. Appl. Cryst.* **53**, 226–235. 10.1107/S1600576719014092 PMC6998782 32047413

[bb39] Marjo, C. E., Bhadbhade, M., Hook, J. M. & Rich, A. M. (2011). *Mol. Pharm.* **8**, 2454–2464. 10.1021/mp200455u 22050389

[bb40] Minkov, V. S., Beloborodova, A. A., Drebushchak, V. A. & Boldyreva, E. V. (2014). *Cryst. Growth Des.* **14**, 513–522.

[bb41] Moreno-Calvo, E., Muntó, M., Wurst, K., Ventosa, N., Masciocchi, N. & Veciana, J. (2011). *Mol. Pharm.* **8**, 395–404. 10.1021/mp100251s 21166472

[bb42] Nowak, M., Dyba, A. J., Janczak, J., Morritt, A., Fábián, L., Karolewicz, B., Khimyak, Y. Z., Braun, D. E. & Nartowski, K. P. (2022). *Mol. Pharm.* **19**, 456–471. 10.1021/acs.molpharmaceut.1c00752 35050637

[bb43] Rigaku Oxford Diffraction (2021). *CrysAlis PRO*. Rigaku Oxford Diffraction, Yarnton, England.

[bb44] Shah, H. S., Chaturvedi, K., Hamad, M., Bates, S., Hussain, A. & Morris, K. (2019). *AAPS PharmSciTech*, **20**, 39–49. 10.1208/s12249-018-1264-0 30604134

[bb45] Sheldrick, G. M. (1996). *SADABS*. University of Göttingen, Germany.

[bb46] Sheldrick, G. M. (2015*a*). *Acta Cryst.* C**71**, 3–8.

[bb47] Sheldrick, G. M. (2015*b*). *Acta Cryst.* A**71**, 3–8.

[bb48] Triggle, A. M., Shefter, E. & Triggle, D. J. (1980). *J. Med. Chem.* **23**, 1442–1445. 10.1021/jm00186a029 6256552

[bb49] Ward, M. R. & Oswald, I. D. H. (2020). *Crystals*, **10**, 1098.

[bb50] Wu, Y., Kobayashi, A., Halder, G. J., Peterson, V. K., Chapman, K. W., Lock, N., Southon, P. D. & Kepert, C. J. (2008). *Angew. Chem. Int. Ed.* **47**, 8929–8932. 10.1002/anie.200803925 18850600

[bb51] Yang, P., Qin, C., Du, S., Jia, L., Qin, Y., Gong, J. & Wu, S. (2019). *Crystals*, **9**, 367–381.

[bb52] Zhou, L., Yin, Q., Du, S., Hao, H., Li, Y., Liu, M. & Hou, B. (2016). *RSC Adv.* **6**, 51037–51045.

