# Supplementary material for: Exploring the thermal behaviour of the solvated structures of nifedipine
Source: Acta Crystallogr B Struct Sci Cryst Eng Mater. 2023 Mar 9;79(Pt 2):164–75. doi: 10.1107/S2052520623001282 (PMC10088478; doi:10.1107/S2052520623001282)
Supplement: Supplementary file 12 [file b-79-00164-sup12.pdf]

Acta Cryst B

STRUCTURAL SCIENCE
CRYSTAL ENGINEERING
MATERIALS

Volume 79 (2023)

Supporting information for article:

Exploring the thermal behaviour of the solvated structures of Nifedipine

Eleanor C. L. Jones, Kate E. Goldsmith, Martin R. Ward, Luis M. Bimbo and Iain D.H. Oswald

Table S1 Unit cell parameters of six identified polymorphs of NIF.

Form	α (Form A) 100 K(Gui *et al.*, 2020)	β (Form C) 100 K	β’ 338 K(Gui *et al.*, 2020)	γ 100 K(Gui *et al.*, 2020)	γ’ 250 K(Gui *et al.*, 2020)	δ 100 K(Gui *et al.*, 2020)
Refcode[a]	BICCIZ07	BICCIZ02	BICCIZ08	BICCIZ09	BICCIZ11	BICCIZ12
a (Å)	10.567(3)	9.6661(6)	9.696(2)	19.065(6)	11.435(4)	11.905(4)
b (Å)	10.408(3)	13.7006(8)	14.231(3)	11.506(4)	12.244(4)	10.908(3)
c (Å)	14.788(4)	14.1184(9)	14.463(3)	15.109(5)	12.327(4)	12.779(4)
α (°)	90	61.028(3)	61.90(3)	90	75.535(16)	90
β (°)	95.028(12)	79.631(4)	80.40(1)	108.962(18)	89.055(16)	106.980(9)
γ (°)	90	81.904(4)	81.80(1)	90	84.774(19)	90
Volume ($Å^3$)	1620.2(8)	1605.89(17)	1731.050	3134.7(18)	1664.2(10)	1587.2(8)
Space Group	$P2_1/c$	$P\bar{1}$	$P\bar{1}$	$P2_1/c$	$P\bar{1}$	$P2_1/n$

[a] Taken from the CSD. (Groom *et al.*, 2016)

Table S2 Solvents used in the solvent screen study of NIF.

Solvent	Grade	Supplier
Acetonitrile	≥99.9%	Fisher
Butyl acetate	≥99.5%	Sigma Aldrich
1,3-dioxane	98.0%	Alfa Aesar
1,4-dioxane	≥99.0%	Alfa Aesar
Dimethylacetamide	≥99.5%	Sigma Aldrich
Dimethylformamide (anhydrous)	99.8%	Sigma Aldrich
Dimethyl sulfoxide	≥99.9%	Sigma Aldrich
Ethanol	99.8%	Fisher

Ethyl Acetate	≥ 99.7%	Sigma Aldrich
Heptane	99.0%	Honeywell
Hexane	≥97.0%	Sigma Aldrich
2-methyltetrahydrofuran	99.0%	Alfa Aesar
Methanol	HPLC	VWR
Morpholine	99.0%	Alfa Aesar
Nitromethane	For synthesis	Merck
2-propanol	ACS Reagent	VWR
Pyridine	≥ 99.0%	Honeywell
Tetrahydrofuran	HPLC ≥ 99.9 %	Honeywell

Table S3 Crystallographic data tables of NIF solvates.

	NIF_14DIO	NIF_MORPH	NIF_THF	NIF_PYRI
Chemical formula	$C_{17}H_{18}N_2O_6{\cdot}C_2H_4O$	$2(C_{17}H_{18}N_2O_6){\cdot}C_4H_9NO$	$2(C_{17}H_{18}N_2O_6){\cdot}C_4H_8O$	$C_{17}H_{18}N_2O_6{\cdot}C_5H_5N$
M_r	390.38	779.79	764.77	425.43
Crystal system, space group	Triclinic, $P\bar{1}$	Triclinic, $P\bar{1}$	Monoclinic, $P2_1/c$	Monoclinic, $P2_1$
Temperature (K)	100	100	100	100
a, *b*, *c* (Å)	7.5604 (1), 11.1362 (1), 11.8563 (1)	7.5423 (6), 11.1513 (9), 11.8923 (9)	13.9233 (14), 9.1421 (9), 14.5374 (15)	9.4363 (10), 14.4247 (14), 15.1387 (15)
α, β, γ (°)	73.606 (1), 73.185 (1), 75.643 (1)	73.997 (2), 73.957 (2), 75.253 (2)	96.280 (4)	96.160 (3)
V (Å^3)	901.54 (2)	906.63 (12)	1839.3 (3)	2048.7 (4)
Z	2	1	2	4
Radiation type	Cu *K*a	Mo *K*a	Mo *K*a	Mo *K*a
μ (mm^{-1})	0.93	0.11	0.11	0.10
Crystal size (mm)	0.4 × 0.35 × 0.25	0.28 × 0.26 × 0.09	0.32 × 0.13 × 0.07	0.32 × 0.24 × 0.09
Diffractometer	XtaLAB Synergy, Single source at home/near, HyPix3000	Bruker *APEX*-II CCD	Bruker *APEX*-II CCD	Bruker *APEX*-II CCD
Absorption correction	Multi-scan *CrysAlis PRO* 1.171.41.99a (Rigaku Oxford Diffraction, 2021) Empirical absorption correction using	Multi-scan *SADABS2016*/2 (Bruker,2016/2) was used for absorption correction. wR2(int) was 0.0990 before and 0.0322 after correction. The Ratio of minimum	Multi-scan *SADABS2016*/2 (Bruker,2016/2) was used for absorption correction. wR2(int) was 0.1461 before and 0.0550 after correction. The Ratio	Multi-scan *SADABS2016*/2 (Bruker,2016/2) was used for absorption correction. wR2(int) was 0.0794 before and

	spherical harmonics, implemented in SCALE3 ABSPACK scaling algorithm.	to maximum transmission is 0.9760. The l/2 correction factor is Not present.	of minimum to maximum transmission is 0.8622. The l/2 correction factor is Not present.	0.0352 after correction. The Ratio of minimum to maximum transmission is 0.9609. The l/2 correction factor is Not present.
T_{min}, T_{max}	0.114, 1.000	0.728, 0.746	0.643, 0.746	0.717, 0.746
No. of measured, independent and observed [$I > 2\sigma(I)$] reflections	21224, 3552, 3543	28798, 4676, 4393	73490, 5614, 4987	54023, 11054, 10804
R_{int}	0.027	0.020	0.036	0.020
$(\sin \theta/\lambda)_{max}$ (Å^{-1})	0.619	0.685	0.715	0.706
$R[F^2 > 2\sigma(F^2)]$, $wR(F^2)$, S	0.036, 0.095, 1.07	0.035, 0.095, 0.97	0.037, 0.102, 1.03	0.029, 0.079, 1.04
No. of reflections	3552	4676	5614	11054
No. of parameters	257	270	275	567
No. of restraints	0	6	5	1
H-atom treatment	H-atom parameters constrained	H atoms treated by a mixture of independent and constrained refinement	H-atom parameters constrained	H-atom parameters constrained
$\Delta\rho_{max}$, $\Delta\rho_{min}$ (e Å^{-3})	0.29, -0.31	0.43, -0.26	0.45, -0.32	0.30, -0.17
Absolute structure	N/A	N/A	N/A	Flack x determined using 4948

				quotients [(I+)-(I-)]/[(I+)+(I-)] (Parsons, Flack and Wagner, Acta Cryst. B69 (2013) 249-259).
Absolute structure parameter	N/A	N/A	N/A	0.20 (9)

Table S4 **Contd.** Crystallographic data tables of NIF solvates.

	NIF_DMSO	NIF_DMA	NIF_DMF	NIF_MeOH
Chemical formula	C2H6OS·C17H18N2O6	C17H18N2O6·C4H9NO	C17H18N2O6·C3H7NO	C17H18N2O6·CH4O
Chemical formula	424.46	433.45	419.43	378.37
Crystal system, space group	Triclinic, $P\bar{1}$	Triclinic, $P\bar{1}$	Triclinic, $P\bar{1}$	Monoclinic, P21/c
Temperature (K)	100	173	100	100
a, *b*, *c* (Å)	7.9036 (10), 11.8798 (15), 11.9713 (15)	7.6638 (10), 11.6006 (16), 13.9001 (19)	7.4526 (13), 11.647 (2), 13.559 (2)	15.6780 (15), 14.4816 (14), 8.0842 (8)
α, β, γ (°)	67.099 (3), 78.341 (3), 79.126 (4)	65.688 (3), 76.979 (3), 92.932 (3)	70.741 (5), 97.785 (5), 73.470 (5)	104.112 (4)
V (Å^3)	1006.5 (2)	1082.7 (3)	1030.4 (3)	1780.1 (3)
Z	2	2	2	4
Radiation type	Mo Ka	Mo Ka	Mo Ka	Mo Ka
μ (mm^{-1})	0.21	0.10	0.10	0.11
Crystal size (mm)	0.16 × 0.05 × 0.01	0.4 × 0.25 × 0.11	0.34 × 0.15 × 0.08	0.40 × 0.23 × 0.08
Diffractometer	Bruker APEX-II CCD	Bruker APEX-II CCD	Bruker APEX-II CCD	Bruker APEX-II CCD
Absorption correction	Multi-scan SADABS2016/2 (Bruker,2016/2) was used for absorption correction. wR2(int) was 0.1110 before and 0.0630 after	Multi-scan SADABS2016/2 (Bruker,2016/2) was used for absorption correction. wR2(int) was 0.1470 before and 0.0676 after	Multi-scan SADABS2016/2 (Bruker,2016/2) was used for absorption correction. wR2(int) was 0.1093 before and 0.0513 after	Multi-scan SADABS2016/2 (Bruker,2016/2) was used for absorption correction. wR2(int) was

	correction. The Ratio of minimum to maximum transmission is 0.7017. The l/2 correction factor is Not present.	correction. The Ratio of minimum to maximum transmission is 0.8046. The l/2 correction factor is Not present.	correction. The Ratio of minimum to maximum transmission is 0.9140. The l/2 correction factor is Not present.	0.1274 before and 0.0492 after correction. The Ratio of minimum to maximum transmission is 0.9302. The l/2 correction factor is Not present.
T_{min}, T_{max}	0.524, 0.746	0.600, 0.746	0.682, 0.746	0.694, 0.746
No. of measured, independent and observed [$I > 2\sigma(I)$] reflections	13422, 5156, 3963	36418, 6605, 5213	35697, 5888, 5050	101200, 5484, 5006
R_{int}	0.054	0.043	0.038	0.032
$(\sin \theta/\lambda)_{max}$ (Å^{-1})	0.705	0.715	0.718	0.717
$R[F^2 > 2\sigma(F^2)]$, $wR(F^2)$, S	0.045, 0.110, 1.02	0.045, 0.130, 1.04	0.037, 0.098, 1.03	0.037, 0.105, 1.03
No. of reflections	5156	6605	5888	5484
No. of parameters	269	309	278	254
No. of restraints	0	13	0	0
H-atom treatment	H-atom parameters constrained	H-atom parameters constrained	H-atom parameters constrained	H atoms treated by a mixture of independent and constrained refinement
$\Delta\rho_{max}$, $\Delta\rho_{min}$ (e Å^{-3})	0.38, −0.56	0.31, −0.23	0.40, −0.27	0.46, -0.39

Computer programs: SAINT,(Bruker, 2018) *CrysAlis PRO* 1.171.41.99a, (Rigaku Oxford Diffraction, 2021)SHELXT,(Sheldrick, 2015*b*) SHELXL,(Sheldrick, 2015*a*) Olex2 1.3 (Dolomanov *et al.*, 2009)

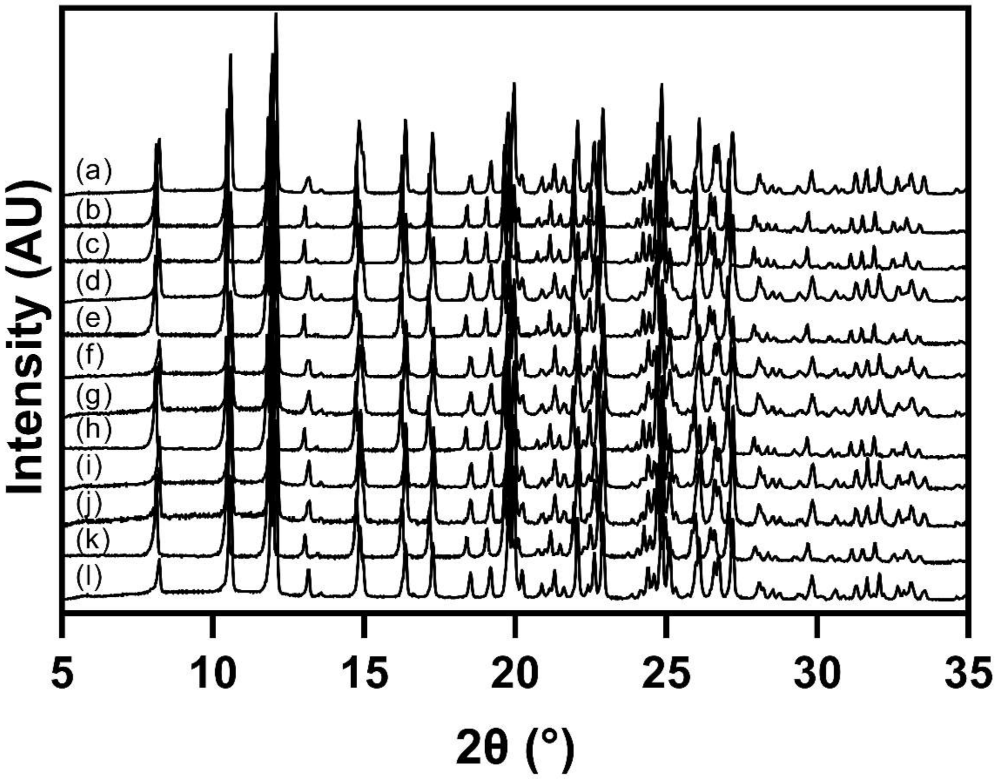

Figure S1 X-ray diffraction pattern of solid material from slurring experiments using: (a) acetonitrile, (b) butyl acetate, (c) 1,3-dioxane, (d) ethanol, (e) ethyl acetate, (f) heptane, (g) hexane, (h) 2-methyltetrahydrofuran, (i) nitromethane, (j) 2-propanol, (k) water and (l) α-NIF as supplied from AlfaAesar.

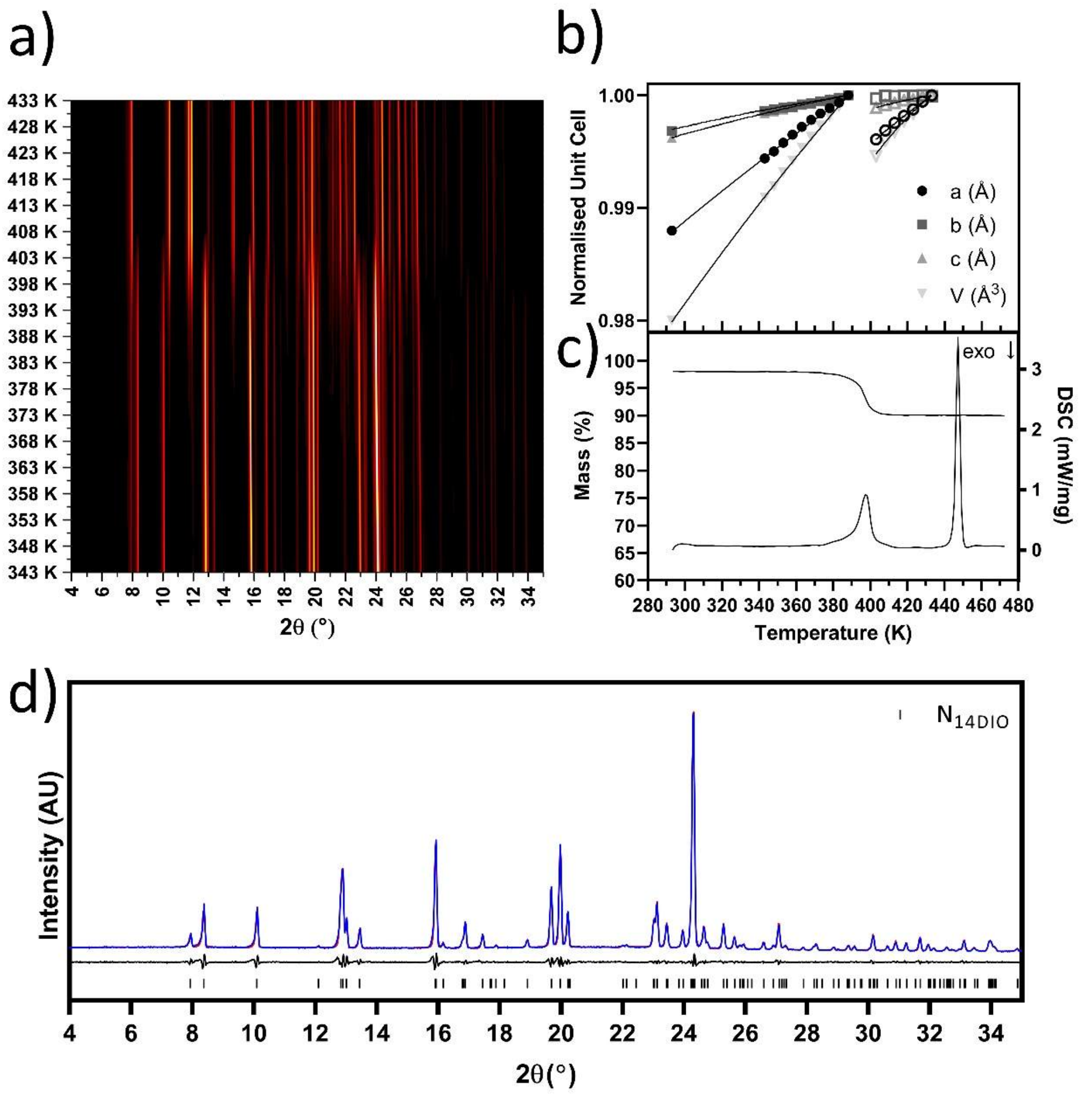

Figure S2 Thermal data of N_{14DIO} capturing the desolvation to the α-form of NIF a) Surface plot of VT-XRPD data from 343 K to 433 K. b) Unit cell parameters from Rietveld refinements of each XRPD pattern. Closed symbols represent data for the solvate, open symbols represent data for desolvated structure. c) DSC and TGA trace for N_{14DIO}. d) XRPD pattern for the 1,4-dioxane solvate collected at 293 K. The experimental data is shown in blue, whilst the calculated profile is shown in red. The difference profile is displayed underneath the diffraction pattern showing that they are in agreement. The calculated reflections are based on the single-crystal data collected for N_{14DIO}.

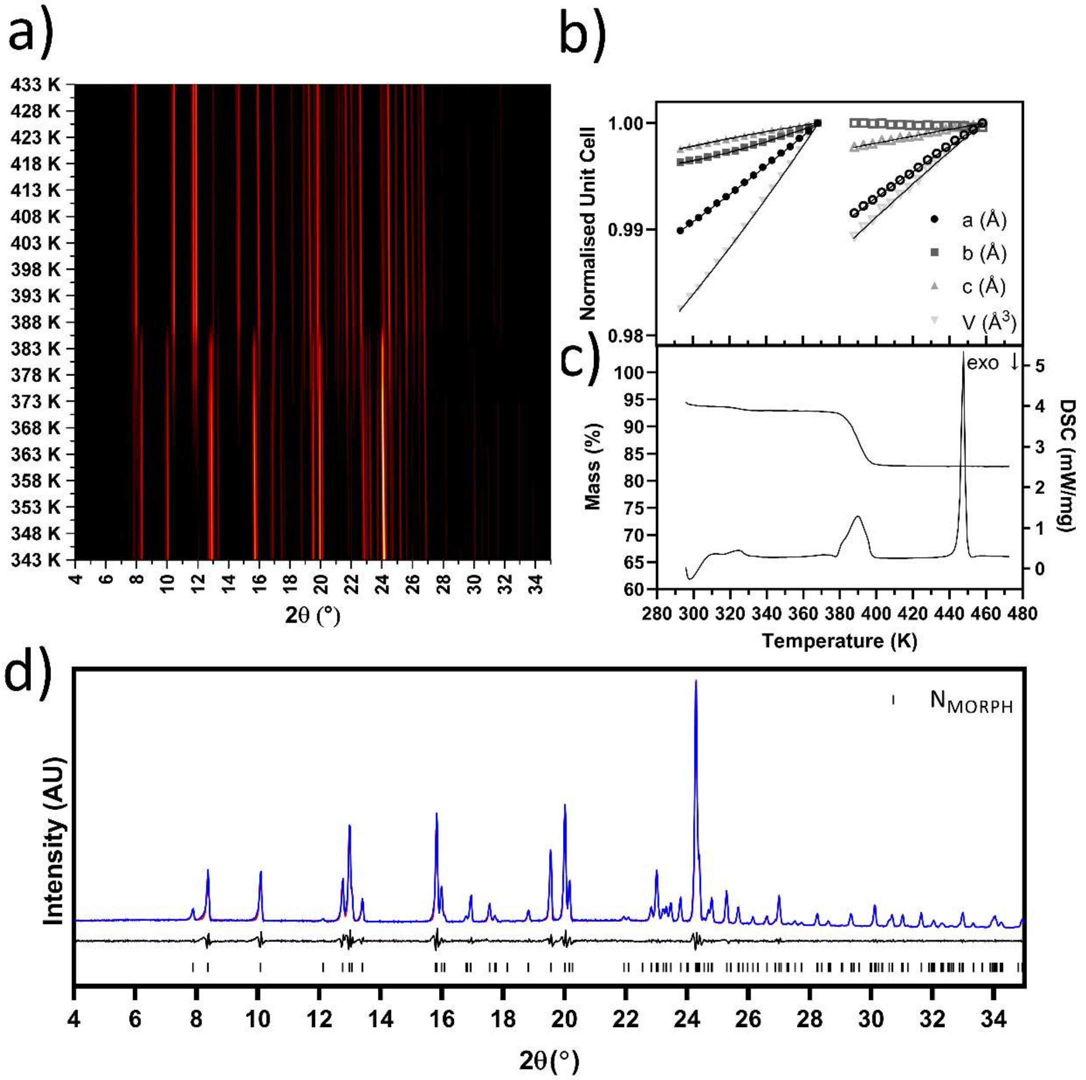

Figure S3 Thermal data of N_{MORPH} capturing the desolvation to the α-form of NIF a) Surface plot of VT-XRPD data from 343 K to 433 K. b) Unit cell parameters from Rietveld refinements of each XRPD pattern. Closed symbols represent data for the solvate; open symbols represent data for desolvated structure. c) DSC and TGA trace for N_{MORPH}. d) XRPD pattern for the morpholine solvate collected at 293 K. The experimental data is shown in blue, whilst the calculated profile is shown in red. The difference profile is displayed underneath the diffraction pattern showing that they are in agreement. The calculated reflections are based on the single-crystal data collected for N_{MORPH}.

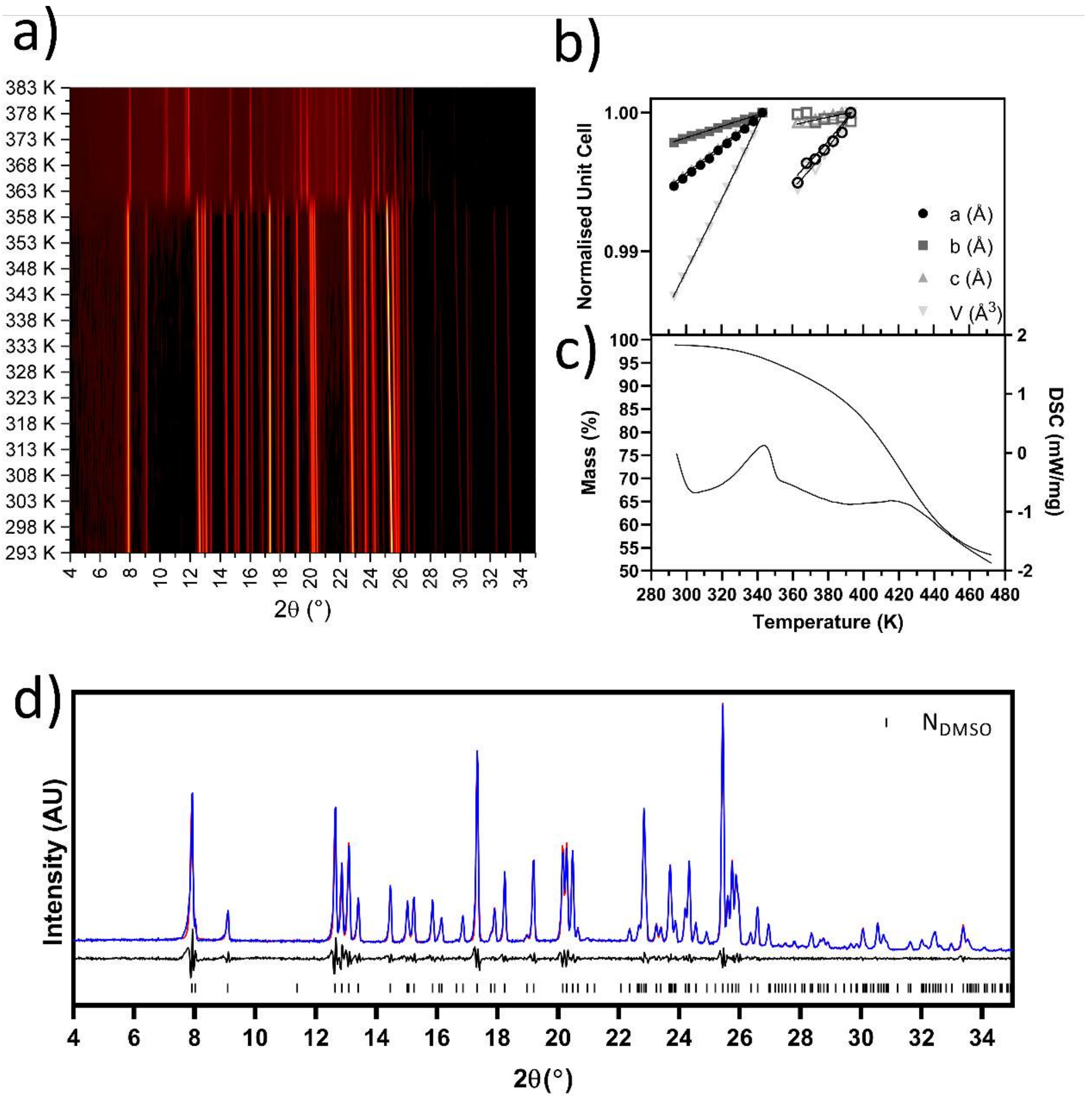

Figure S4 Thermal data of NDMSO capturing the desolvation to α-NIF a) Surface plot of VT-XRPD data from 293 K to 383 K. b) Unit cell parameters from Rietveld refinements of each XRPD pattern. The data could only be fitted to a temperature of 393 K. Closed symbols represent data for the solvate; open symbols represent data for desolvated structure. c) DSC and TGA trace for NDMSO. No prominent melting event is witnessed. d) XRPD pattern for the DMSO solvate collected at 293 K. The experimental data is shown in blue, whilst the calculated profile is shown in red. The difference profile is displayed underneath the diffraction pattern. The calculated reflections are based on the single-crystal data collected for NDMSO.

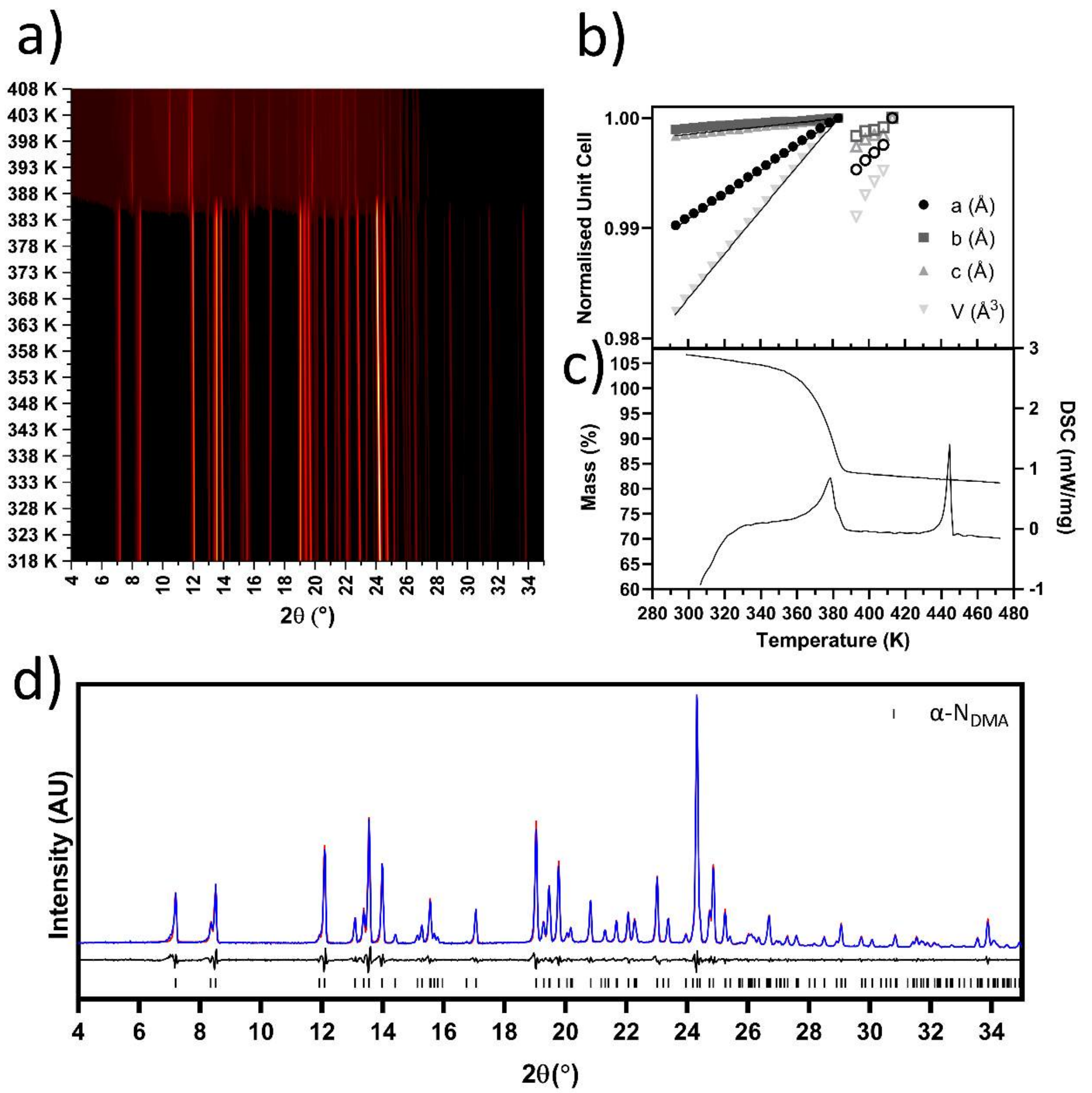
a)
b)
c)
d)
408 K
403 K
398 K
393 K
388 K
383 K
378 K
373 K
368 K
363 K
358 K
353 K
348 K
343 K
338 K
333 K
328 K
323 K
318 K
2θ (°)
Normalised Unit Cell
a (Å)
b (Å)
c (Å)
V (Å3)
Mass (%)
DSC (mW/mg)
Temperature (K)
Intensity (AU)
α-NDMA
2θ(°)

Figure S5 Thermal data of α-N_{DMA} capturing the desolvation to α-NIF a) Surface plot of VT-XRPD data from 293 K to 403 K. Melt of the sample is captured from 388 K. b) Unit cell parameters from Rietveld refinements of each XRPD pattern. The data could only be fitted to a temperature of 413 K. Closed symbols represent data for α-N_{DMA}; open symbols represent data for β-N_{DMA} structure. c) DSC and TGA trace for N_{DMA}. d) XRPD pattern for the DMA solvate collected at 293 K. The experimental data is shown in blue, whilst the calculated profile is shown in red. The difference profile is displayed underneath the diffraction pattern. The calculated reflections are based on the single-crystal data collected for α-N_{DMA}.

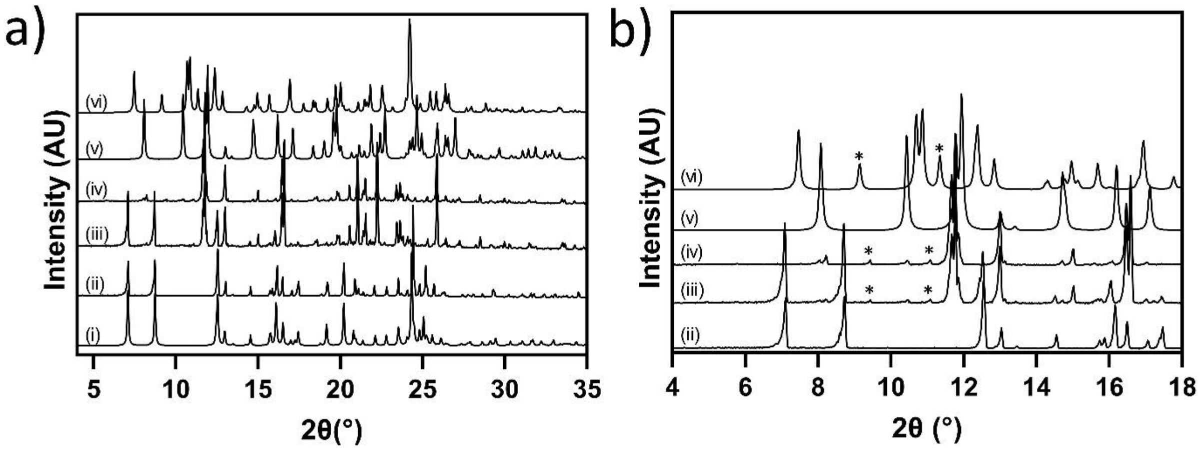

Figure S6 a) XRPD of NDMF solvate from 4 to 35°, 2θ. (i) Simulated powder pattern from SC-data. VT patterns at (ii) 293 K; (iii) 353 K and (iv) 358 K. The latter temperatures show the start of desolvation to α-NIF. (v) Simulated powder pattern of α-NIF from SC-data collected at 297 K and (vi) Powder pattern of β-NIF collected at 296 K (BICCIZ03). b) Expanded view of Figure a, highlighting the region where a form other than α-NIF appears during desolvation and 353 and 358K. Asterisks indicate reflections that might correspond to Form β-NIF but that are significantly shifted in 2-theta.

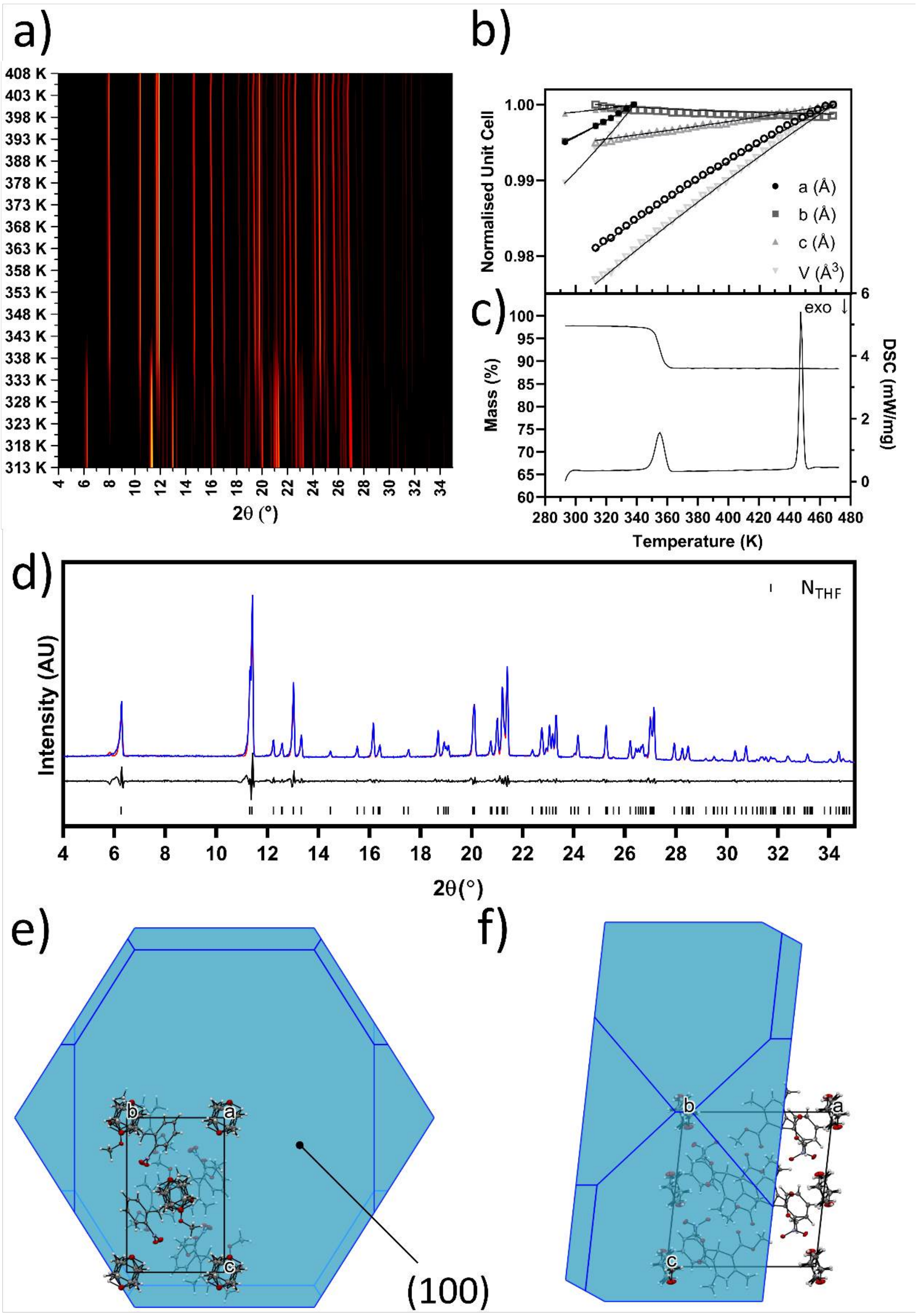

Figure S7 Thermal data of N_{THF} capturing the desolvation to the α-form of NIF a) Surface plot of VT-XRPD data from 313 K to 408 K. b) Unit cell parameters from Rietveld refinements of each XRPD pattern. Closed symbols represent data for the solvate; open symbols represent data for desolvated structure. c) DSC and TGA trace for N_{THF}. d) XRPD pattern for the THF solvate collected

at 293 K. The experimental data is shown in blue, whilst the calculated profile is shown in red. The difference profile is displayed underneath the diffraction pattern showing that they are in agreement. The calculated reflections are based on the single-crystal data collected for N_{THF}. The BFDH morphology for NTHF along e) a-axis and f) b-axis indicating the placement of the THF molecules with respect to the largest face of the crystal. The location of the THF molecules facilitates the loss on heating.

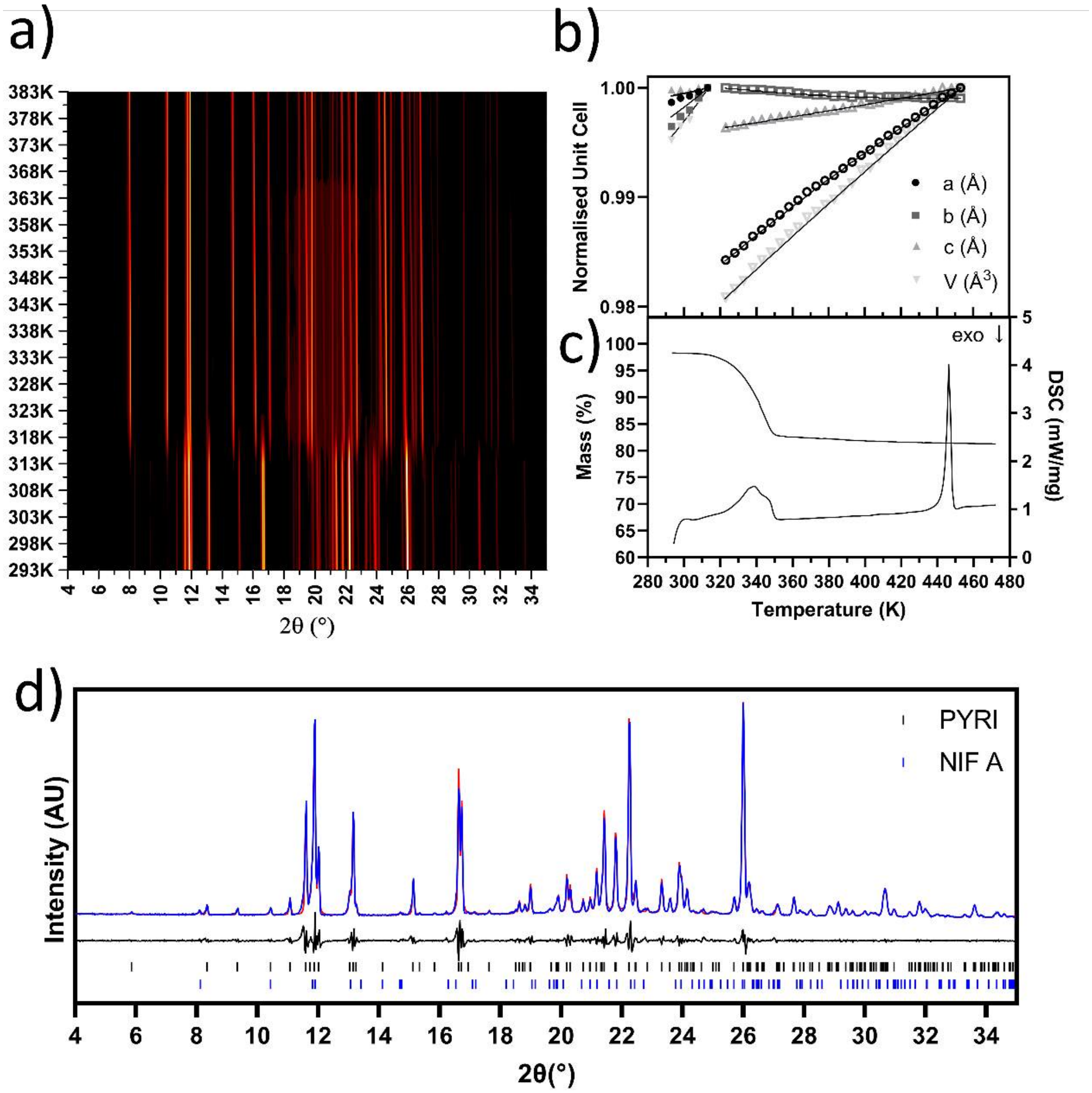

Figure S8 Thermal data of N_{PYRI} capturing the desolvation to the α-form of NIF a) Surface plot of VT-XRPD data from 293 K to 383 K. b) Unit cell parameters from Pawley refinements of each XRPD pattern. Closed symbols represent data for the solvate; open symbols represent data for desolvated structure. c) DSC and TGA trace for N_{PYRI}. d) XRPD pattern for the pyridine solvate collected at 293 K. The experimental data is shown in blue, whilst the calculated profile is shown in red. The difference profile is displayed underneath the diffraction pattern. The calculated reflections are based on the single-crystal data collected for N_{PYRI} and NIF α-form.

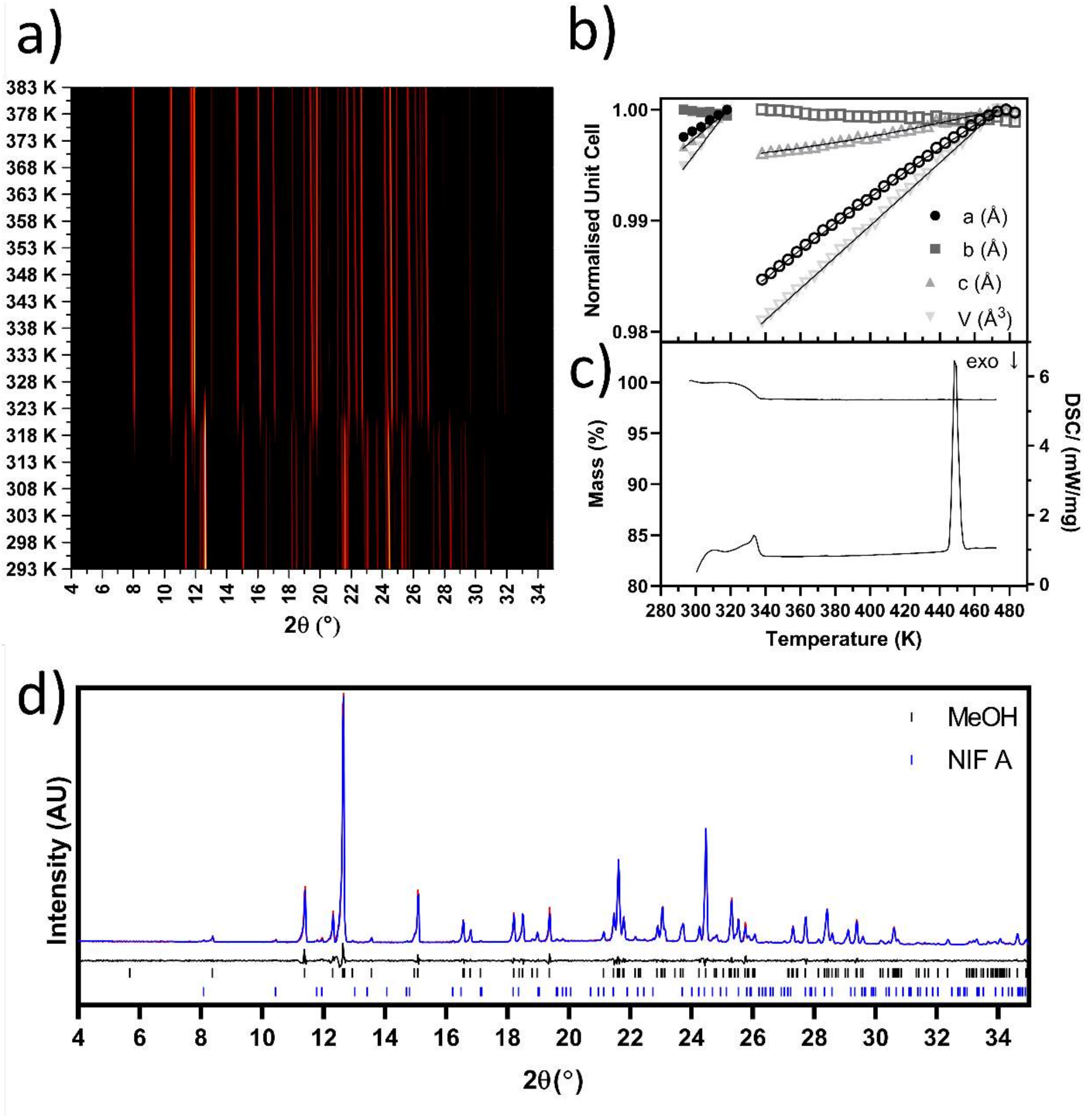

Figure S9 Thermal data of N_{MeOH} capturing the desolvation to α-NIF a) Surface plot of VT-XRPD data from 293 K to 383 K. b) Unit cell parameters from Pawley refinements of each XRPD pattern. Closed symbols represent data for the solvate; open symbols represent data for desolvated structure. c) DSC and TGA trace for N_{MeOH}. d) XRPD pattern for the DMF solvate collected at 293 K. The experimental data is shown in blue, whilst the calculated profile is shown in red. The difference profile is displayed underneath the diffraction pattern. The calculated reflections are based on the single-crystal data collected for N_{MeOH}. The solvate is only formed from slow evaporation hence the single-crystals were ground for both VT-XRPD and STA. The pulverisation of single-crystals may have induced mechanical desolvation as there was α-NIF present in all of the diffraction patterns as small trace quantities.

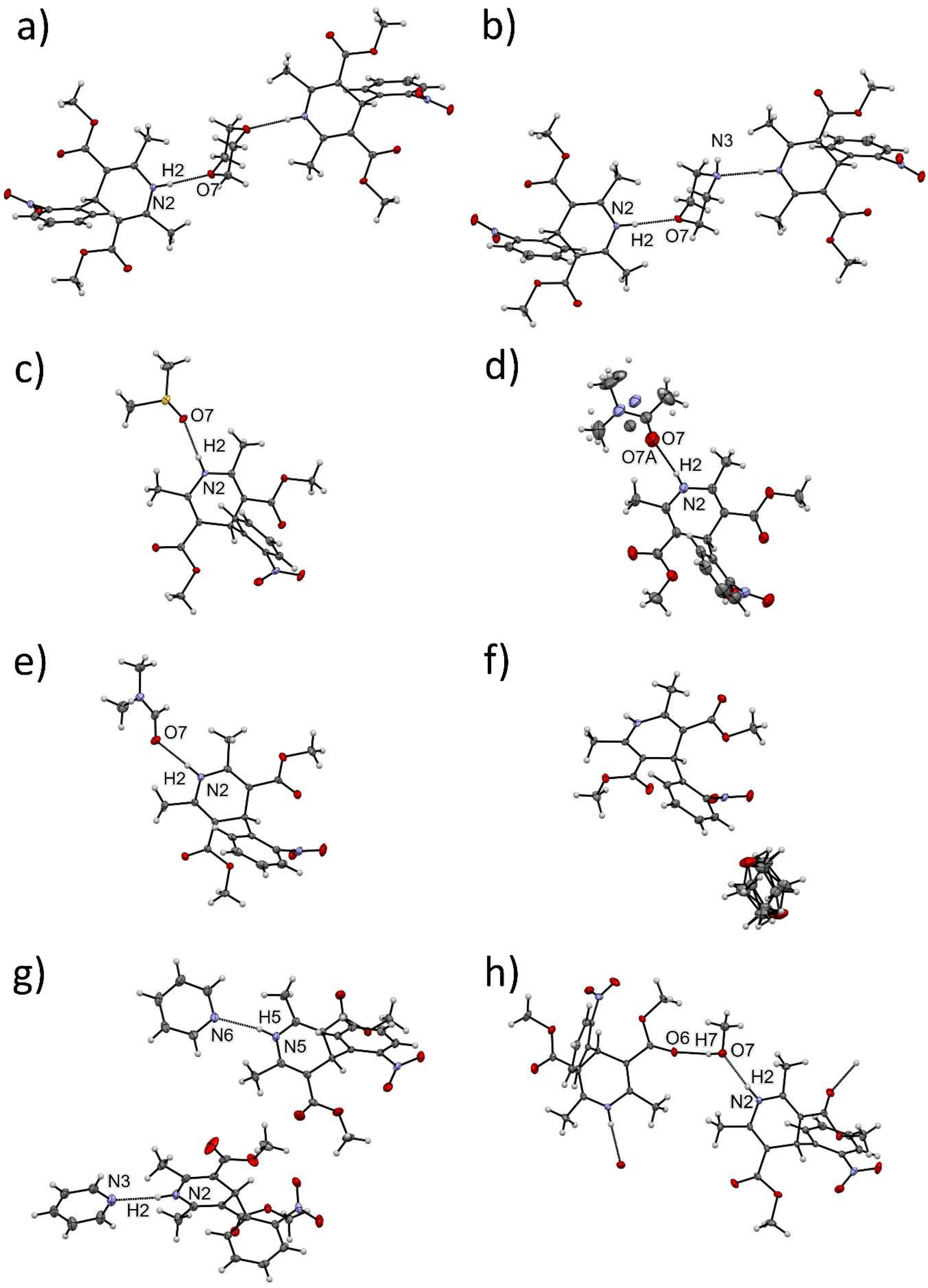

Figure S10 Interactions between NIF and solvents: a) 1,4-dioxane; b) morpholine; c) DMSO; d) DMA (which shows disorder); e) DMF; f) THF (note hydrogen bonding is absent between solvent and

NIF molecule); g) pyridine and h) methanol. Labelled atoms show those which are involved in hydrogen bonds.
