# Supplementary figures and images for: Exploring the thermal behaviour of the solvated structures of nifedipine
Source: Acta Crystallogr B Struct Sci Cryst Eng Mater. 2023 Mar 9;79(Pt 2):164–75. doi: 10.1107/S2052520623001282 (PMC10088478; doi:10.1107/S2052520623001282)

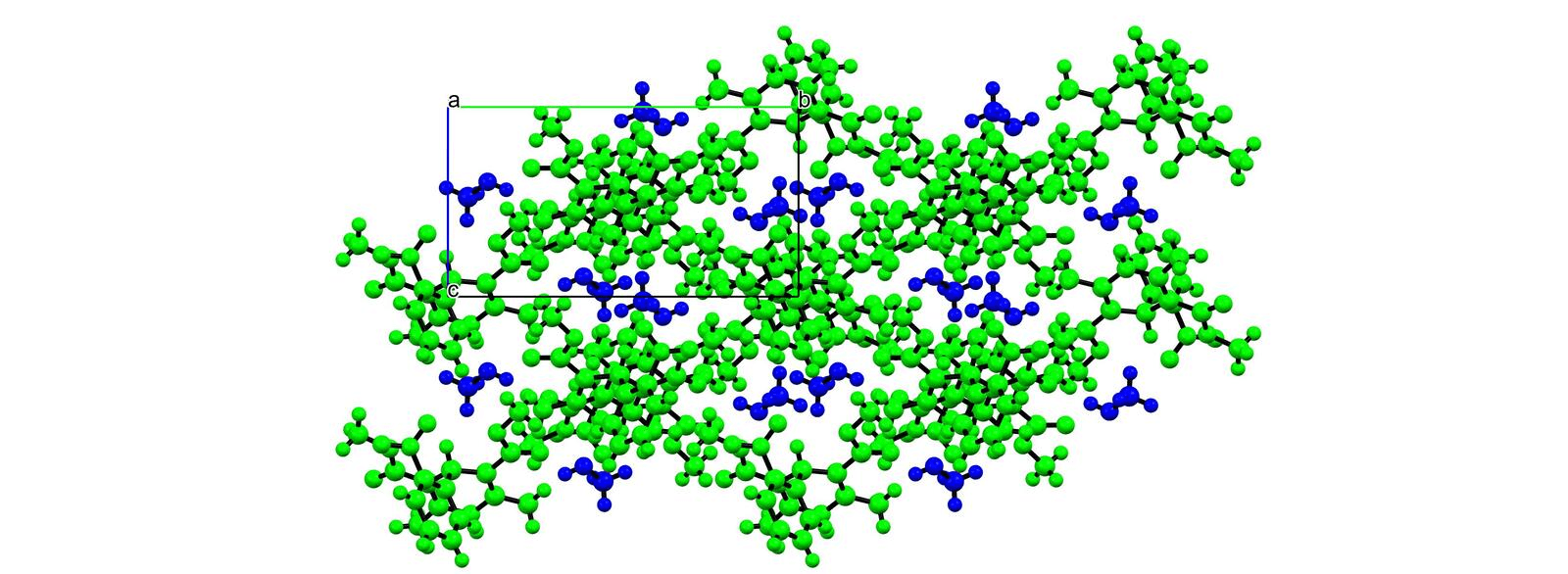

Supplement: Supplementary file 13 [file b-79-00164-sup13.gif]
